# Titanium tetrafluoride catalysis for the dehydrative conversion of diphenylmethanols to symmetric and unsymmetric ethers†

**DOI:** 10.1039/d4ra04712e

**Published:** 2024-08-02

**Authors:** Aman G. Singh, Abdulkhaliq A. Alawaed, P. Veeraraghavan Ramachandran

**Affiliations:** a Department of Chemistry, Purdue University West Lafayette IN 47907 USA chandran@purdue.edu

## Abstract

In contrast to the conversion of diphenylmethanol to the corresponding halides with an equivalent of titanium tetrachloride or -bromide, catalytic (50 mol%) titanium tetrafluoride converts benzhydrols in diethyl ether or dichloromethane to bis(benzhydryl) ethers within 0.5–1 h at room temperature. Cross ether formation with diphenylmethanols and primary aryl or aliphatic alcohols is achieved in the presence of 25 mol% TiF_4_ in refluxing toluene as solvent. A tentative mechanism involving a carbocation intermediate has been proposed.

## Abstract 2

Catalytic titanium tetrafluoride efficiently converts benzhydrols to bisbenzhydryl ethers or cross-ethers (with primary alcohols) within 0.5–2 h, achieving high yields.

## Graphical abstract

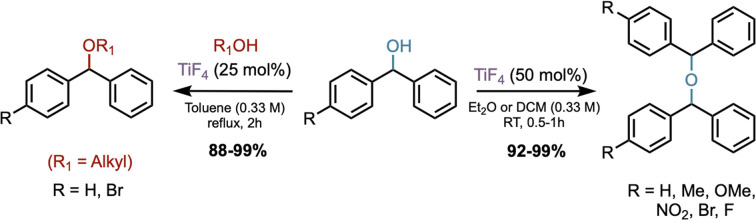

## Introduction

**Fig. 1:**
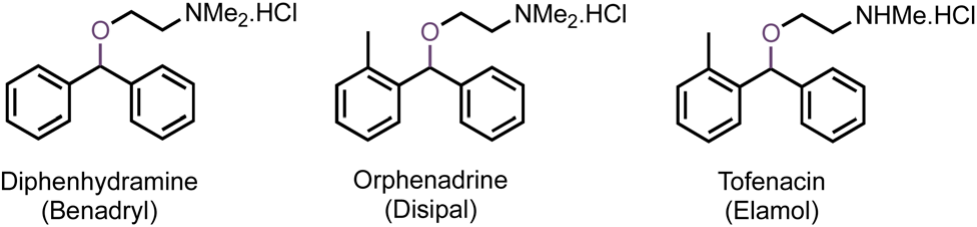
Diphenylmethyl aminoethyl ether pharmaceuticals.

The formation of diphenylmethyl ethers (DPME) from alcohols and their *trans*-etherification have been subjects of investigation for several decades due to the interest in DPME protection of alcohols during multi-step organic syntheses.^1^ In addition, DPMEs are an integral part of several pharmacologically important molecules, such as the antihistamine diphenhydramine (benzhydryl dimethylaminoethyl ether) hydrochloride (Benadryl^®^),^2^ anti-cholinergic orphenadrine hydrochloride (Disipal^®^),^3^ anti-depressant tofenacin hydrochloride (Elamol^®^),^3^*etc.* (Fig. 1). Several procedures for the direct self- and cross-etherification of benzyl alcohols, particularly diphenylmethanol have been reported in the literature.^4,5^ The preparation of DPMEs reported eight decades ago used tri-diphenylmethylphosphate as an alkylating agent, accelerated using trifluoracetic acid as a catalyst.^6^ Bis(diphenylmethyl) ether was also prepared using (diethylamino)sulfur trifluoride (DAST),^7^ zeolite,^8^ or *p*-toluenesulfonyl chloride (*p*-TsCl),^9^*etc.* as catalysts. Cross ethers from DPM and alcohols can be prepared employing several catalysts, such as Fe(NO_3_)_3,_^10^ Fe(OTf)_3_,^11^ Cu(NO_3_)_2_,^12^ MoO_2_(acac)_2_,^13^ PdCl_2_,^14–16^ BF_3_–Et_2_O,^17^ Nafion-H,^18^ NaAuCl_4_,^19^*etc.* Use of diphenyldiazomethane^20^ and trichloroacetimidate^21^ for the synthesis of DPMEs and *trans*-etherification of DPMEs with ytterbium triflate [Yb(OTF)_3_]^22^ and FeCl_3_ ^23^ have also been reported.

**Scheme 1:**
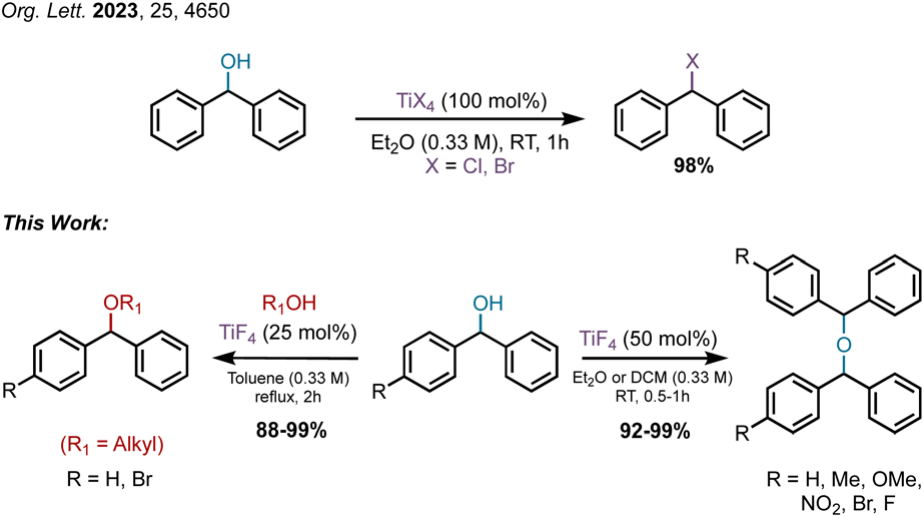
Reactions of diphenylmethanol with/in the presence of TiX_4_.

Our accidental discovery of the etherification of diphenylmethanols in the presence of titanium tetrafluoride (TiF_4_) originated from the reduction of benzophenone to diphenylmethanol (DPM, 1a) with borane-ammonia in the presence of titanium tetrachloride (TiCl_4_).^24^ We had observed that in addition to DPM, the corresponding diphenylmethyl chloride could also be prepared by altering the stoichiometry of TiCl_4_.^25^ This led to a titanium tetrachloride or -bromide-mediated conversion of benzhydrols to benzydryl halides (Scheme 1),^25^ which serve as precursors for several piperazine derivatives possessing biological properties.^26^ This dehydroxyhalogenation was extended to benzyl alcohol and other alcohols as well.^25^ We had postulated that the halogenation of DPM and alcohols proceeds *via* a carbocation intermediate and, indeed, recently reported on the use of benzyl alcohols as pre-electrophiles for Friedel–Crafts reactions in the presence of TiCl_4_.^27^ Based on a reported titanium tetrafluoride-mediated fluorination during Prins cyclization,^28^ we were interested in examining the potential for a dehydroxyfluorination of alcohols using TiF_4_. Unexpectedly, the reaction of DPM with a molar equiv. of TiF_4_ in diethyl ether (Et_2_O) at room temperature (RT) resulted in the formation of the corresponding bis(benzhydryl) ether (2a) in 91% yield within 30 minutes. Further examination of this reaction has led to an efficient dehydrative dimerization of substituted DPMs and cross-etherification with primary alcohols. An examination of the plausible mechanism of this reaction was also undertaken.

## Results and discussion

The effect of stoichiometry, solvent, concentration, *etc.* on the TiF_4_-mediated room-temperature self-etherification was assessed first (Table 1). Optimization of the catalyst stoichiometry revealed that 50 mol% of TiF_4_ is sufficient to complete the dehydrative dimerization. The reaction was very facile at RT in Et_2_O, dichloromethane (DCM), and hexanes. A reaction in toluene at RT gave the bis(diphenylmethyl) ether 2a and the Friedel–Crafts product 5 in an 84 : 16 ratio (*vide infra*). The reaction in other solvents, such as tetrahydrofuran (THF), and nitromethane show product formation, but fail to undergo completion (TLC). Solvents such as dimethoxyethane (DME) and acetonitrile do not facilitate self-etherification, probably due to complexation with the catalyst.^29^ The solubility of the catalyst in the solvents was not favourable for a higher concentration reaction and optimal yields were achieved in 0.33 M Et_2_O, DCM, and *n*-hexane. The best yields were obtained when using DCM as solvent.

**Table 1:** Optimization of reaction conditions for the preparation of 2a from 1a in the presence of catalytic TiF_4_ at RT^a^

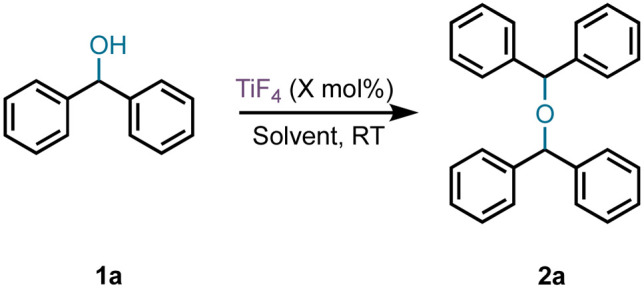				
Entry	TiF_4_, mol%	Solvent	Reaction time, h	bProduct 1a : 2a (yield%)
1	10	Et_2_O	24	57 : 43
2	25	Et_2_O	24	25 : 75
3	50	Et_2_O	0.5	0 : 100 (91)
4	50	Hexanes	0.5	0 : 100 (85)
5	50	DCM	1	0 : 100 (97)
6	50	Toluene	2	0 : 84 : 16^c^
7	50	CH_3_CN	0.5	100 : 0
8	50	DME	0.5	100 : 0
9	50	THF	1	95 : 5
10	50	NO_2_Me	1	22 : 78

a All reactions were carried out at 1 mmol scale with 0.33 M solvent.

b Isolated yields.

c Friedel–Crafts reaction product.

Having standardized the reaction, a series of diphenylmethanols, prepared *via* the sodium borohydride reduction of the corresponding benzophenones or Grignard reaction of the corresponding benzaldehydes bearing an electron-donating and -with-drawing substituent on the phenyl ring, were converted to the corresponding bis(benzydryl) ethers in Et_2_O or CH_2_Cl_2_. Thus, DPMs with a 4-bromo- (1b), 4-methoxy- (1c), 4-methyl- (1d), 4-nitro- (1e), and 4-fluoro-(1f) substituent on one of the phenyl rings were converted to the bis-ethers 2b–2f in 92–99% yields (Table 2). DPMs substituted with electron-withdrawing groups and halogens provided the corresponding bis-ethers in near quantitative yields. However, those with electron-donating groups provided slightly lower yields. Evidently, this may be attributed to the stability of the intermediate carbocations (*vide infra*).

**Table 2:** Preparation of bis(diphenylmethyl) ethers in the presence of catalytic TiF_4_ at room temperature^a^

Entry	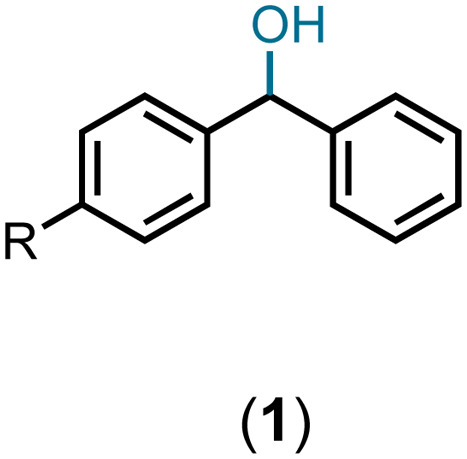		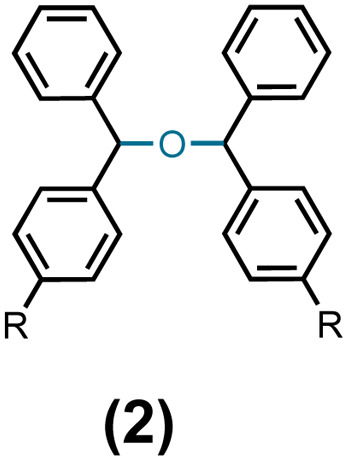		
#	#	R	#	Solvent	bYield%
1	1a	H	2a	DCM	97
2	1b	Br	2b	DCM	99
3	1c	OMe	2c	Et_2_O	95
4	1d	Me	2d	Et_2_O	92
5	1e	NO_2_	2e	DCM	99
6	1f	F	2f	Et_2_O	99

a Reaction at 1 mmol scale with 0.33 M Et_2_O/CH_2_Cl_2_ at RT in the presence of 50 mol% TiF_4_.

b Isolated yields.

Curious whether benzyl alcohol (3a) can be converted to dibenzyl ether in the presence of TiF_4_, a reaction was performed in Et_2_O at RT. Unlike the reaction of 3a with titanium tetrachloride and -bromide which led to the corresponding benzyl halides,^25^ the reaction with TiF_4_ did not yield any of the fluoride nor the corresponding dibenzyl ether products; the alcohol was recovered completely.

We sought to exploit this lack of reactivity of a primary alcohol to develop a direct cross-etherification/protection of alcohols by preparing the DPM ether *via* TiF_4_ catalysis. Unfortunately, a reaction of 1a and 3a in diethyl ether in the presence of 50 mol%, or even 100 mol% TiF_4_ resulted only in the formation of 2a and none of the cross ether (4aa). Fortuitously, when the above reaction was performed in the presence of 25 mol% TiF_4_ at higher temperature, in refluxing toluene, 4aa was isolated in 91% yield within 2 h. Notably, not even traces of 2a were observed during this reaction. To verify whether the formation of 4aa is proceeding *via* a *trans*-etherification of 2a,^11^ a solution of 2a and 3a in toluene was refluxed for 2 h, with and without TiF_4_. None of 4aa was formed in the latter reaction, but the former reaction revealed the formation of 4aa, albeit at a slow rate. The reversibility of the bis-ether formation step is discussed later (*vide infra*: mechanism). A similar reaction with methanol (3b) in refluxing toluene provided 96% of the cross ether (4ab) and none of the dimer 2a (Table 3).

**Table 3:** Preparation of alkyl (diphenylmethyl) ethers in the presence of catalytic TiF_4_^a^

#	1	ROH (3)		Ether (4)		
		#	R	#	Structure	bYield%
1	1a	3a	Bn	4aa	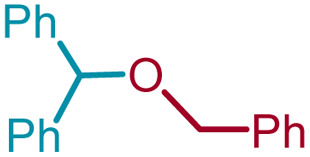	91
2	1a	3b	Me	4ab	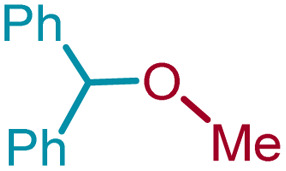	96^c^
3	1b	3b	Me	4bb	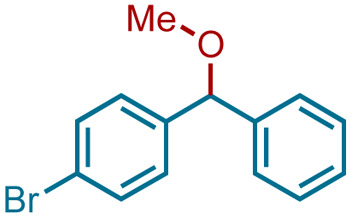	99^c^
4	1a	3c	Et	4ac	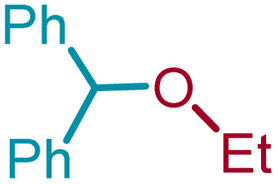	91^c^
5	1a	3d	*n*-Bu	4ad	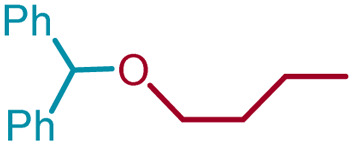	96
6	1a	3e	ClCH_2_CH_2_	4ae	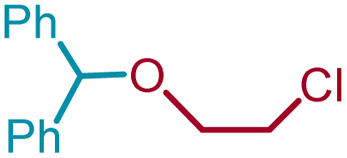	91
7	1a	3f	*p*-ClBn	4af	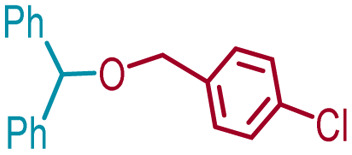	98
8	1a	3g	*n*-C_6_H_11_	4ag	NR^d^	
9	1a	3h	*t*-Bu	4ah	NR^d^	
10	1a	3i	CH_2_ <svg xmlns="http://www.w3.org/2000/svg" version="1.0" width="13.200000pt" height="16.000000pt" viewBox="0 0 13.200000 16.000000" preserveAspectRatio="xMidYMid meet"><metadata> Created by potrace 1.16, written by Peter Selinger 2001-2019 </metadata><g transform="translate(1.000000,15.000000) scale(0.017500,-0.017500)" fill="currentColor" stroke="none"><path d="M0 440 l0 -40 320 0 320 0 0 40 0 40 -320 0 -320 0 0 -40z M0 280 l0 -40 320 0 320 0 0 40 0 40 -320 0 -320 0 0 -40z"/></g></svg> CHCH_2_	4ai	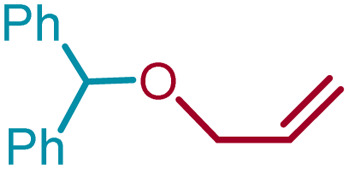	94^c^
11	1b	3i	CH_2_CHCH_2_	4bi	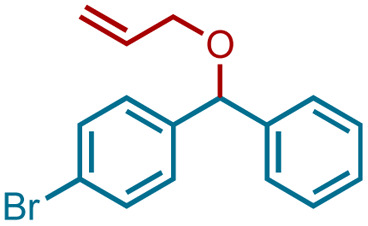	97^c^

a Reaction conditions: 1 mmol scale, reflux in 0.33 M toluene for 2 h with 25 mol% TiF_4_.

b Isolated yields.

c With 1.25 equiv. of 3.

d NR = no reaction.

Ethanol (3c), and *n*-butanol (3d) provided the corresponding ethers 4ac and 4ad, in 91% and 96% yields, respectively. Similarly, 4-bromo-substituted benzhydrol (1b) provided the corresponding methoxy ether (4bb) in 99% yield. 2-Chloroethanol (3e) and *p*-chlorobenzyl alcohol (3f) were also treated with 1a, which provided high yields of 91% and 98% respectively for the corresponding DPM ethers, 4ae and 4af, respectively. Chloroether 4ae is an intermediate for the preparation of Benadryl^®^.^2^ More hindered 2°- and 3°-alcohols, cyclohexanol (3g) and tert-butanol (3h), respectively failed to provide the desired etherification products 4ag and 4ah respectively in toluene as solvent, but 2a was formed. On the other hand, allyl alcohol (3i) when reacted with the DPMs 1a and 1b yielded 94% and 97% of ethers 4ai and 4bi, respectively.

## Reaction mechanism

**Scheme 2:**
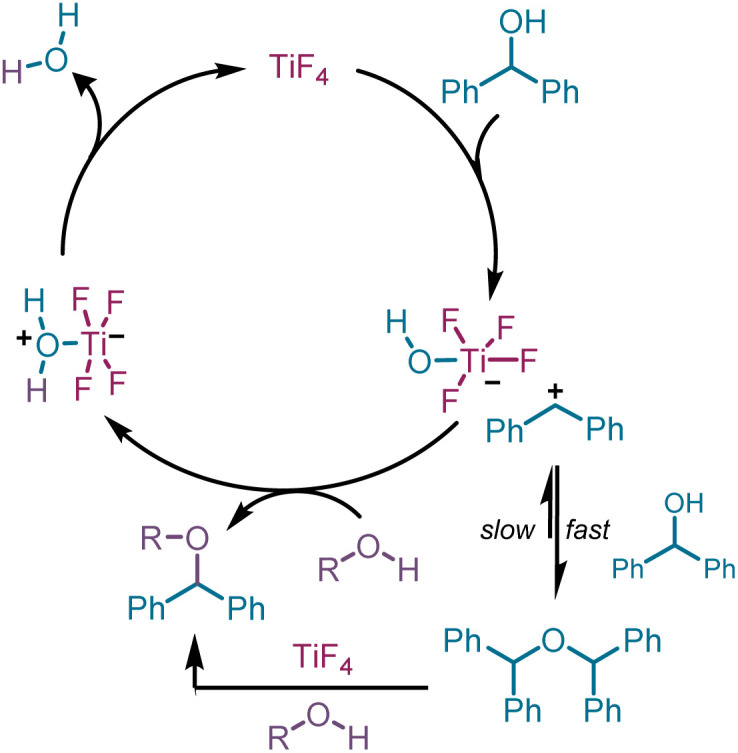
Mechanistic pathway for TiF_4_-mediated etherification of DPM.

Having developed efficient protocols for the preparation of symmetrical and unsymmetrical ethers from DPMs, we turned our attention to rationalize the difference in behaviour of the tetrafluoro-reagent compared to the tetrachloro- and tetrabromotitanium derivatives. We had earlier established that the chlorination and bromination occurs *via* a carbocation,^25^ which was confirmed by carrying out a Friedel–Crafts reaction with pro-electrophiles, such as alcohols in the presence of the latter reagents.^27^ It is known that alcohols and amines form a complex with titanium tetrafluoride.^29^ Once this occurs, an S_N_1 pathway can be envisaged for the formation of the ether involving an intermediate carbocation (Scheme 2).

The intermediacy of the carbocation can be presumed from the Friedel–Crafts alkylation product during the reaction of 1a in toluene as solvent at RT (Table 1, entry 6). Indeed, to demonstrate the presence of the carbocation unambiguously, a Friedel–Crafts reaction of DPM and an equivalent of TiF_4_ was conducted in refluxing benzene, anticipating the formation of triphenylmethane (5). The reaction proceeded to completion in 2 h and the ^1^H NMR of the product revealed the formation of 5 along with 2a in a 2 : 1 ratio. To facilitate the Friedel–Crafts alkylation, we carried out a similar reaction with DPM and 50 mol% TiF_4_ in refluxing toluene, which is a better substrate for Friedel–Crafts due to the increased electron density of the phenyl ring. Indeed, we isolated (*p*-tolylmethylene)dibenzene (5) exclusively in 96% yield, confirming the presence of a carbocation intermediate (Table 4). It is noteworthy that the triphenylmethane moiety forms the backbone for several dyes,^30,31^ and drugs possessing antiseptic,^32^ antihelmintic, and antimicrobial properties.^33^ They are also present in photodynamic therapy^34^ agents.

**Table 4:** TiF_4_-catalyzed Friedel–Crafts reaction^a^

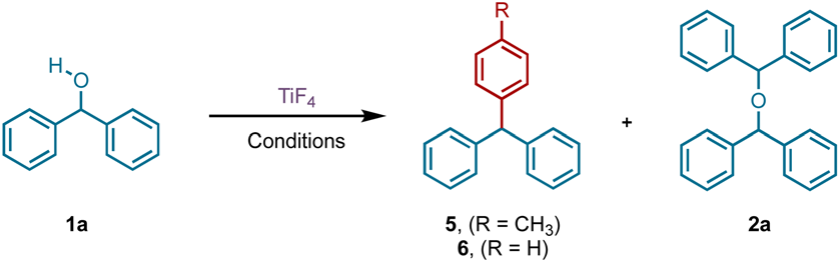					
Entry	Product		Reaction conditions		
	#	5/6 : 2a (yield %)	Solvent	Temp.	TiF_4_ mol%
1	5	16 : 84	Toluene	RT	50
2	5	100 : 0 (96)	Toluene	Reflux	50
3	6	66 : 34	Benzene	Reflux	100

a Reactions carried out at 1 mmol scale.

## Conclusion

In conclusion, we have developed a facile titanium tetrafluoride-catalysed dehydration protocol for the synthesis of symmetric and unsymmetric ethers from diphenylmethanol and related compounds by themselves at RT or with primary alcohols in refluxing toluene. This quick, room temperature synthesis of symmetrical ethers affords yields in the range of 92–99% and the cross-ethers in refluxing toluene in 91–99% yields. Mechanistic studies point to a carbocation pathway, which is confirmed by a TiF_4_-mediated Friedel–Crafts reaction. Although the process is efficient in preparing ethers, it fails when amines are used as the nucleophile, perhaps due to the complexation of TiF_4_ with amines. Continued studies on a potential dehydrative amination are underway.

## Data availability

The data supporting this article have been included as part of the ESI.†

## Author contributions

P. V. Ramachandran: funding acquisition, conceptualization, project administration, writing – review and editing; A. G. Singh: data curation, investigation, methodology, validation; A. A. Alawaed: data curation, investigation, methodology, validation.

## Conflicts of interest

There are no conflicts to declare.

## Supplementary Material

RA-014-D4RA04712E-s001
