# Supplementary material for: Titanium tetrafluoride catalysis for the dehydrative conversion of diphenylmethanols to symmetric and unsymmetric ethers
Source: RSC Adv. 2024 Aug 2;14(33):24236–9. doi: 10.1039/d4ra04712e (PMC11296350; doi:10.1039/d4ra04712e)
Supplement: RA-014-D4RA04712E-s001 [file RA-014-D4RA04712E-s001.pdf]

Supplementary Information (SI) for RSC Advances.
This journal is © The Royal Society of Chemistry 2024

SUPPORTING INFORMATION:

Titanium tetrafluoride catalysis for the dehydrative conversion of diphenylmethanols to symmetric and unsymmetric ethers

Aman G. Singh, Abdulkhaliq A. Alawaed, and P. Veeraraghavan Ramachandran*

† Herbert C. Brown Center for Borane Research, Department of Chemistry, Purdue University, West Lafayette, Indiana 47907, United States

E-mail: chandran@purdue.edu

Contents:

Page

General information……………………………………………………………………S2

General procedure for the preparation of homodimer ether…………………………….……..S2

General procedure for the preparation of unsymmetrical ethers compounds..…………………. S3

Experimental Section:

General Information:

All reagents and starting materials were purchased from Sigma-Aldrich and Oakwood and used as received. Anhydrous Et_2O was distilled from sodium-benzophenone and stored under nitrogen atmosphere. Thin layer chromatography (TLC) was performed on silica gel F60 plates and visualized under UV light or ceric ammonium molybdate solution. The products were confirmed by nuclear magnetic resonance (NMR) spectroscopy and measured in δ values in parts per million (ppm) relative to tetramethylsilane (TMS) as the internal standard (TMS: 0.00 ppm). ^1H NMR spectra were recorded from Bruker 400 MHz spectrometer or Bruker 300 MHz spectrophotometer at ambient temperature and calibrated against the residual solvent peak of $CDCl_3$ (δ = 7.26 ppm) as an internal standard. The ^{13}C NMR spectra were reported at 101 MHz (297 K) or 75 MHz (297 K) and calibrated using $CDCl_3$ (δ = 77.0 ppm) as an internal standard. Coupling constants (J) are given in hertz (Hz), and signal multiplicities are described of NMR data as s = singlet, d = doublet, t = triplet, dd = double doublet, dt = double triplet, q= quartet, p= pentet, m = multiplet, and br = broad. ^{19}F NMR spectra

A. General procedure for the preparation of homodimer ethers (2a-2f):

A 50 mL oven dried round bottom flask was charged with TiF_4 (0.5mmol, 0.5eq) and a magnetic stirring bar. The flask was sealed using a rubber septum. Diethyl ether or methylene chloride (3 mL) was added, and subsequently the benzhydrol (1.0 mmol, 1.0 eq) was added to the solution. Upon complete addition the reaction flask was again sealed with a septum. The reaction mixture was allowed to stir at room temperature and monitored by TLC until completion. On completion of the reaction (~0.5-1h), the crude mixture was then transferred to a separatory funnel and extracted with 3M NaOH (2 × 10mL) and 3M HCl (1 x 10mL) using methylene chloride as the organic phase. The combined organic layers were dried over anhydrous sodium sulfate, filtered through cotton, and concentrated under rotary evaporation and the remaining solvent was removed by applying a high vacuum for 2 hours.

B. General procedure for the preparation of unsymmetrical ethers (4aa-4bi)

A 50 mL oven dried round bottom flask was charged with TiF_4 (0.5mmol, 0.5eq) and a magnetic stirring bar. The flask was sealed using a rubber septum. Toluene (3mL) was added, and subsequently diphenylmethanol (1.0mmol, 1.0eq) was added to the solution. A reflux condenser was attached to the flask, and the reaction mixture was brought to reflux using an oil bath and monitored with TLC. On completion of the reaction (~2hrs), the crude mixture was then transferred to a separatory funnel and extracted with 3M NaOH (2 × 10mL) and 3M HCl (1 x 10mL) using methylene chloride as the organic phase. The combined organic layers were dried over anhydrous sodium sulfate, filtered through cotton, and concentrated under rotary evaporation and the remaining solvent was removed by applying a high vacuum for 2 hours.

Characterization of Products:

(oxybis(methanetriyl))tetrabenzene (2a)

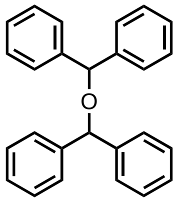

Colorless oil, mass = 0.342g, 97% yield

The compound was prepared as described in the general procedure **A**.
^{1}H NMR (300 MHz, $CDCl_3$) δ 7.3 – 7.2 (m, 20H), 5.3 (s, 2H).
^{13}C {^{1}H} NMR (75 MHz, $CDCl_3$) δ 141.2, 127.3, 126.4, 126.2, 79.0.
Characterisation is in agreement with previous reports of this compound.[1]

4,4'-(oxybis(phenylmethylene))bis(bromobenzene) (2b)

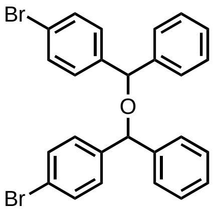

White solid, mass = 0.253g, 99% yield

The compound was prepared as described in the general procedure **A**.
^{1}H NMR (300 MHz, $CDCl_3$) δ 7.51 – 7.38 (m, 4H), 7.37 – 7.18 (m, 14H), 5.32 (s, 2H).
^{13}C {^{1}H} NMR (75 MHz, $CDCl_3$) δ 141.3, 141.2, 141.1, 141.0, 131.6, 131.5, 128.9, 128.8, 128.6, 128.55, 127.9, 127.8, 127.2, 127.1, 121.5, 79.6, 53.4.
Characterisation is in agreement with previous reports of this compound.[2]

4,4'-(oxybis(phenylmethylene))bis(methoxybenzene) (2c)

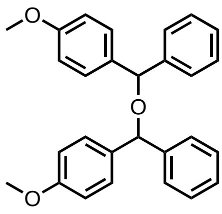

Colorless amorphous solid, mass = 0.231g, 91% yield

The compound was prepared as described in the general procedure **A**.
^{1}H NMR (400 MHz, $CDCl_3$) δ 7.39 (d, J = 8.4 Hz, 4H), 7.34 (t, J = 7.6 Hz, 4H), 7.32 – 7.23 (m, 6H), 6.88 (d, J = 8.8 Hz, 4H), 5.38 (s, 2H), 3.80 (s, 6H).
^{13}C {^{1}H} NMR (101 MHz, $CDCl_3$) δ 158.9, 142.6, 142.4, 134.4, 134.2, 128.6, 128.5, 128.3, 128.2, 127.2, 127.1, 127.0, 113.72, 113.68, 79.4, 55.2.
Characterisation is in agreement with previous reports of this compound.[3]

4,4'-(oxybis(phenylmethylene))bis(methylbenzene) (2d)

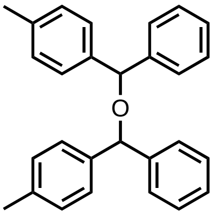

Colorless oil, mass = 0.174g, 92% yield

The compound was prepared as described in the general procedure **A**.
^{1}H NMR (400 MHz, $CDCl_3$) δ 7.36 (d, *J* = 6.7 Hz, 4H), 7.30 (t, *J* = 7.7 Hz, 4H), 7.24 (d, *J* = 8.5 Hz, 6H), 7.12 (d, *J* = 7.8 Hz, 4H), 5.36 (s, 2H), 2.32 (s, 6H).
^{13}C {^{1}H} NMR (101 MHz, $CDCl_3$) δ 142.6, 142.4, 139.3, 139.1, 137.0 , 139.96, 129.0, 128.3, 127.2, 127.2, 127.1, 79.7, 21.1.
Characterisation is in agreement with previous reports of this compound [3].

4,4'-(oxybis(phenylmethylene))bis(nitrobenzene) (2e)

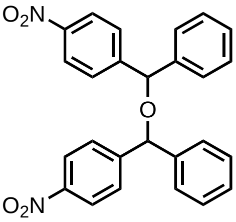

Yellow oil, mass = 0.228g, 99% yield

The compound was prepared as described in the general procedure **A**.
^{1}H NMR (300 MHz, $CDCl_3$) δ 8.17 (td, *J* = 9.1, 1.8 Hz, 4H), 7.55 (ddd, *J* = 9.0, 7.3, 1.8 Hz, 4H), 7.42 – 7.28 (m, 10H), 5.48 (s, 2H).
^{13}C {^{1}H} NMR (75 MHz, $CDCl_3$) δ 149.3, 148.8, 147.4, 147.2, 140.1, 139.7, 129.1, 128.9, 128.7, 128.4, 127.7, 127.5, 127.4, 127.1, 123.9, 123.7, 79.8, 79.7.
Characterisation is in agreement with previous reports of this compound.[3]

4,4'-(oxybis(phenylmethylene))bis(fluorobenzene) (2f)[4]

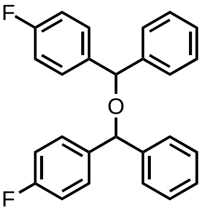

Colorless oil, mass = 0.191g, 99% yield

The compound was prepared as described in the general procedure **A**.
^{1}H NMR (400 MHz, $CDCl_3$) δ 7.38 – 7.23 (m, 14H), 7.07 – 6.99 (m, 4H), 5.35 (s, 2H).
^{13}C {^{1}H} NMR (101 MHz, $CDCl_3$) δ 141.7, 137.8, 128.7, 128.4, 127.5, 127.1, 127.0, 115.3, 115.1, 79.4.
^{19}F NMR (376 MHz, $CDCl_3$) δ -115.0.
HRMS (ESI+) m/z: [M+H]+ calculated for $C_{26}H_{21}F_2O$: 387.1555, found 387.1580.

((benzyloxy)methylene)dibenzene (4aa)

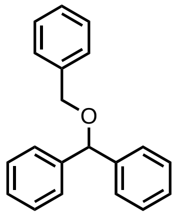

Yellow oil, mass = 0.249g, 91% yield

The compound was prepared as described in the general procedure **B**.
^{1}H NMR (400 MHz, $CDCl_3$) δ 7.4 – 7.3 (m, 15H), 5.5 (s, 1H), 4.6 (s, 2H).
^{13}C {^{1}H} NMR (101 MHz, $CDCl_3$) δ 142.1, 138.3, 128.3, 128.3, 127.6, 127.4, 127.4, 127.0, 82.4, 70.4.
Characterisation is in agreement with previous reports of this compound. [1]

(methoxymethylene)dibenzene (4ab)

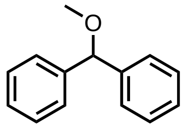

Colorless liquid, mass = 0.191g, 96.3% yield

The compound was prepared as described in the general procedure **B**.
^{1}H NMR (300 MHz, $CDCl_3$) δ 7.46 – 7.18 (m, 10H), 5.24 (s, 1H), 3.38 (s, 3H).
^{13}C {^{1}H} NMR (75 MHz, $CDCl_3$) δ 142.1, 128.4, 127.5, 127.3, 126.9, 85.4, 57.0
Characterisation is in agreement with previous reports of this compound.[5]

1-bromo-4-(methoxy(phenyl)methyl)benzene (4bb)

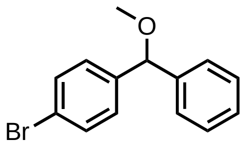

Colorless liquid, mass = 0.276g, 99% yield

The compound was prepared as described in the general procedure **B**.
^{1}H NMR (300 MHz, $CDCl_3$) δ 7.46 (dd, *J* = 8.3, 1.8 Hz, 2H), 7.39 – 7.19 (m, 7H), 5.20 (s, 1H), 3.38 (s, 3H).
^{13}C {^{1}H} NMR (75 MHz, $CDCl_3$) δ 141.5, 141.2, 131.5, 128.6, 128.5, 127.7, 126.9, 84.7, 57.0
Characterisation is in agreement with previous reports of this compound.[6]

(ethoxymethylene)dibenzene (4ac)

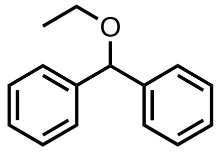

Colorless liquid, mass = 0.194g, 91.4% yield

The compound was prepared as described in the general procedure **B**.
^{1}H NMR (300 MHz, $CDCl_3$) δ 7.48 – 7.19 (m, 10H), 5.38 (s, 1H), 3.54 (q, *J* = 7.0 Hz, 2H), 1.37 – 1.22 (m, 3H).
^{13}C {^{1}H} NMR (75 MHz, $CDCl_3$) δ 142.6, 128.4, 127.3, 127.0, 83.5, 64.5, 15.3.
Characterisation is in agreement with previous reports of this compound.[5]

(butoxymethylene)dibenzene (4ad)

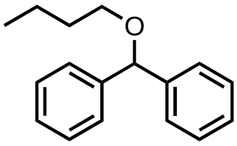

Colorless liquid, mass = 0.230g, 95.7% yield

The compound was prepared as described in the general procedure **B**.
^{1}H NMR (300 MHz, $CDCl_3$) δ 7.38 – 7.18 (m, 10H), 5.32 (s, 1H), 3.44 (t, *J* = 6.5 Hz, 2H), 1.71 – 1.56 (m, 2H), 1.50 – 1.33 (m, 2H), 0.90 (t, *J* = 7.3 Hz, 3H).
^{13}C {^{1}H} NMR (75 MHz, $CDCl_3$) δ 142.7, 128.3, 127.3, 127.0, 83.6, 68.9, 32.0, 19.5, 14.0.
Characterisation is in agreement with previous reports of this compound.[1]

((2-chloroethoxy)methylene)dibenzene (4ae)

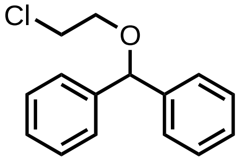

Colorless liquid, mass = 0.225g, 91.2% yield

The compound was prepared as described in the general procedure **B**.
^{1}H NMR (300 MHz, $CDCl_3$) δ 7.36 (d, *J* = 0.9 Hz, 10H), 5.44 (s, 1H), 3.79 – 3.64 (m, 4H).
^{13}C {^{1}H} NMR (75 MHz, $CDCl_3$) δ 141.7, 128.5, 127.6, 127.0, 84.0, 69.1, 43.0
Characterisation is in agreement with previous reports of this compound.[7]

(((4-chlorobenzyl)oxy)methylene)dibenzene (4af)

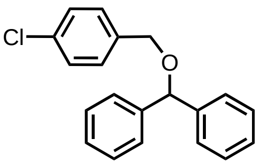

Colorless liquid, mass = 0.303g, 98.1% yield
The compound was prepared as described in the general procedure **B**.
^{1}H NMR (300 MHz, $CDCl_3$) δ 7.43 – 7.22 (m, 14H), 5.43 (s, 1H), 4.51 (s, 2H).
^{13}C {^{1}H} NMR (75 MHz, $CDCl_3$) δ 141.9, 129.0, 128.5, 128.5, 127.6, 127.1, 82.7, 69.8.
Characterisation is in agreement with previous reports of this compound.[8]

((allyloxy)methylene)dibenzene (4ai)

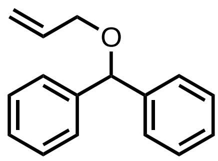

Colorless liquid, mass = 0.210g, 93.6% yield

The compound was prepared as described in the general procedure **B**.
^{1}H NMR (300 MHz, $CDCl_3$) δ 7.42 – 7.19 (m, 10H), 5.97 (ddt, *J* = 17.2, 10.4, 5.5 Hz, 1H), 5.42 (s, 1H), 5.30 (dq, *J* = 17.2, 1.7 Hz, 1H), 5.19 (dq, *J* = 10.4, 1.5 Hz, 1H), 4.01 (dt, *J* = 5.5, 1.5 Hz, 2H).
^{13}C {^{1}H} NMR (75 MHz, $CDCl_3$) δ 142.2, 134.8, 128.4, 127.4, 127.0, 116.9, 82.6, 69.7.
Characterisation is in agreement with previous reports of this compound.[1]

1-((allyloxy)(phenyl)methyl)-4-bromobenzene (4bi)

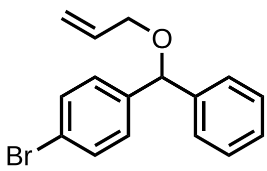

Yellow liquid, mass = 0.294g, 96.9% yield

The compound was prepared as described in the general procedure **B**.
^{1}H NMR (300 MHz, $CDCl_3$) δ 7.49 – 7.42 (m, 2H), 7.37 – 7.21 (m, 7H), 5.97 (ddt, *J* = 17.2, 10.4, 5.5 Hz, 1H), 5.38 (s, 1H), 5.31 (dq, *J* = 17.2, 1.7 Hz, 1H), 5.21 (ddd, *J* = 10.4, 2.2, 1.0 Hz, 1H), 4.01 (d, *J* = 5.5 Hz, 2H).
^{13}C {^{1}H} NMR (75 MHz, $CDCl_3$) δ 141.6, 141.4, 134.5, 131.5, 128.7, 128.5, 127.7, 127.0, 121.3, 117.1, 81.9, 69.7
Characterisation is in agreement with previous reports of this compound.[5]

(*p*-tolylmethylene)dibenzene (5)

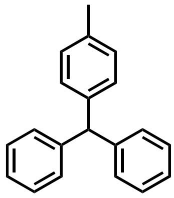

Colorless liquid, mass = 0.248g, 96% yield

The compound was prepared as described in the general procedure
^{1}H NMR (300 MHz, $CDCl_3$) δ 7.35 – 6.98 (m, 14H), 5.53 (s, 1H), 2.33 (s, 3H).
^{13}C {^{1}H} NMR (75 MHz, $CDCl_3$) δ 144.1, 140.9, 135.7, 129.4, 129.3, 129.0, 128.2, 126.2, 56.4, 21.0
Characterisation is in agreement with previous reports of this compound.[9]

References:

1. V. H. Tran, M. T. La and H.-K. Kim, *Organic & Biomolecular Chemistry*, 2019, **17**, 6221-6228.
2. P. K. Sahoo, S. S. Gawali and C. Gunanathan, *ACS Omega*, 2018, **3**, 124-136.
3. A. Kumar, A. K. Pal, R. D. Anand, T. V. Singh and P. Venugopalan, *Tetrahedron*, 2011, **67**, 8308-8313.
4. *No spectral data available for this compound in literature.*
5. J. Li, X. Zhang, H. Shen, Q. Liu, J. Pan, W. Hu, Y. Xiong and C. Chen, *Advanced Synthesis & Catalysis*, 2015, **357**, 3115-3120.
6. H. Hu, S.-J. Chen, M. Mandal, S. M. Pratik, J. A. Buss, S. W. Krska, C. J. Cramer and S. S. Stahl, *Nature Catalysis*, 2020, **3**, 358-367.
7. J. L. Richardson, I. R. E. Nett, D. C. Jones, M. H. Abdille, I. H. Gilbert and A. H. Fairlamb, *ChemMedChem*, 2009, **4**, 1333-1340.
8. Q. Xu, H. Xie, P. Chen, L. Yu, J. Chen and X. Hu, *Green Chemistry*, 2015, **17**, 2774-2779.
9. Y. Xia, F. Hu, Z. Liu, P. Qu, R. Ge, C. Ma, Y. Zhang and J. Wang, *Organic Letters*, 2013, **15**, 1784-1787.

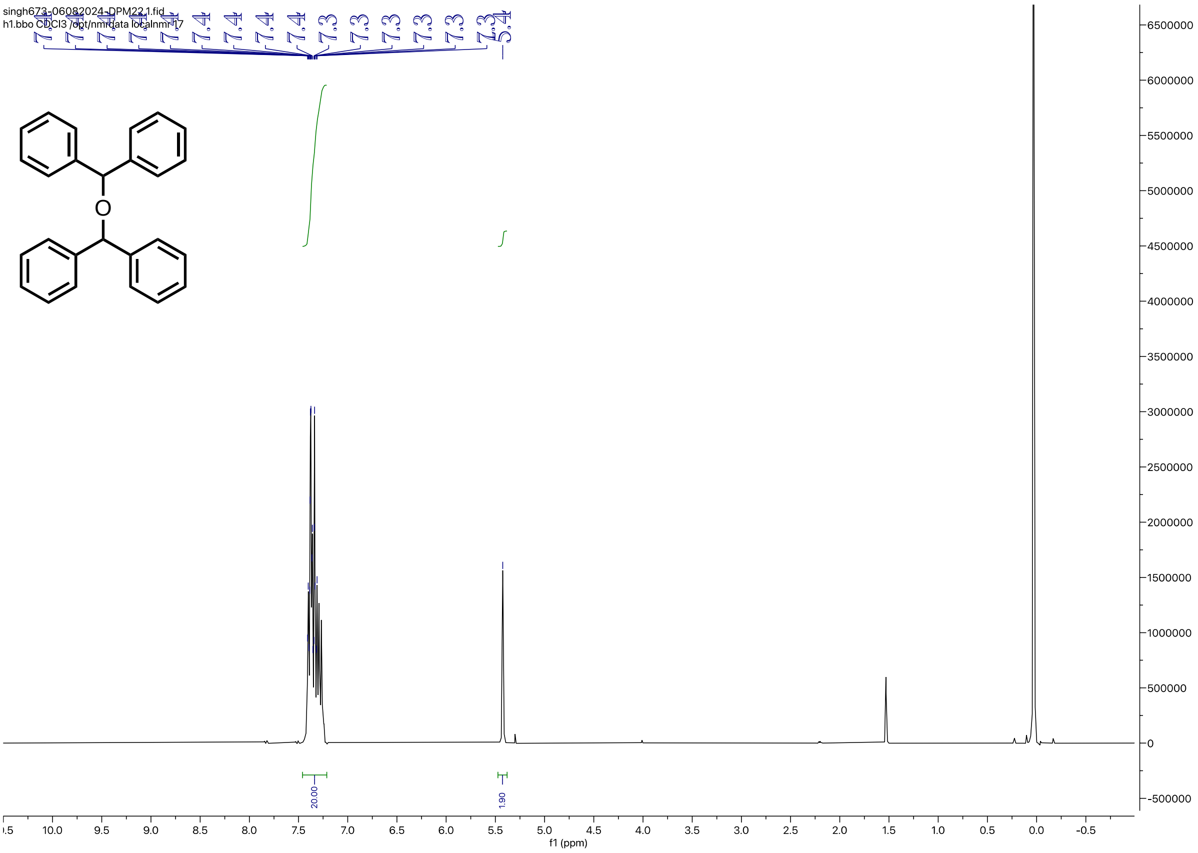

^{1}H NMR (300 MHz, Chloroform-*d*) (oxybis(methanetriyl))tetrabenzene **(2a)**

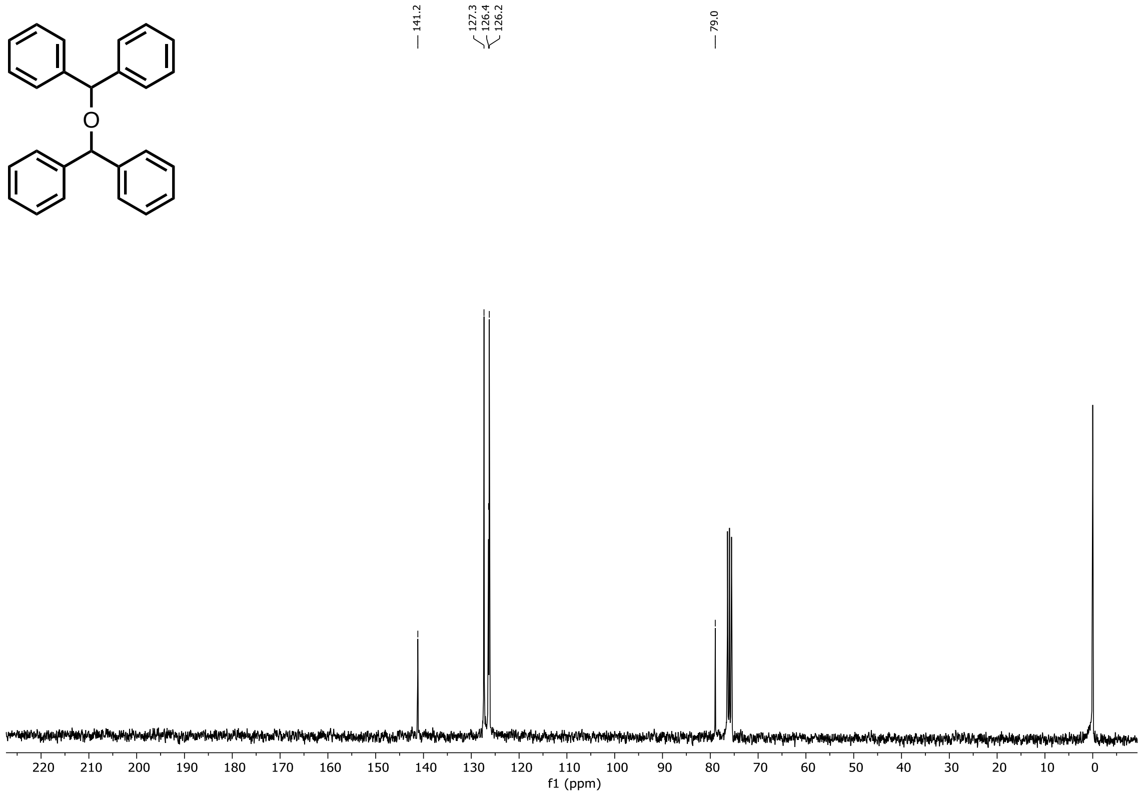

^{13}C {^{1}H} NMR (75 MHz, Chloroform-*d*) (oxybis(methanetriyl))tetrabenzene **(2a)**

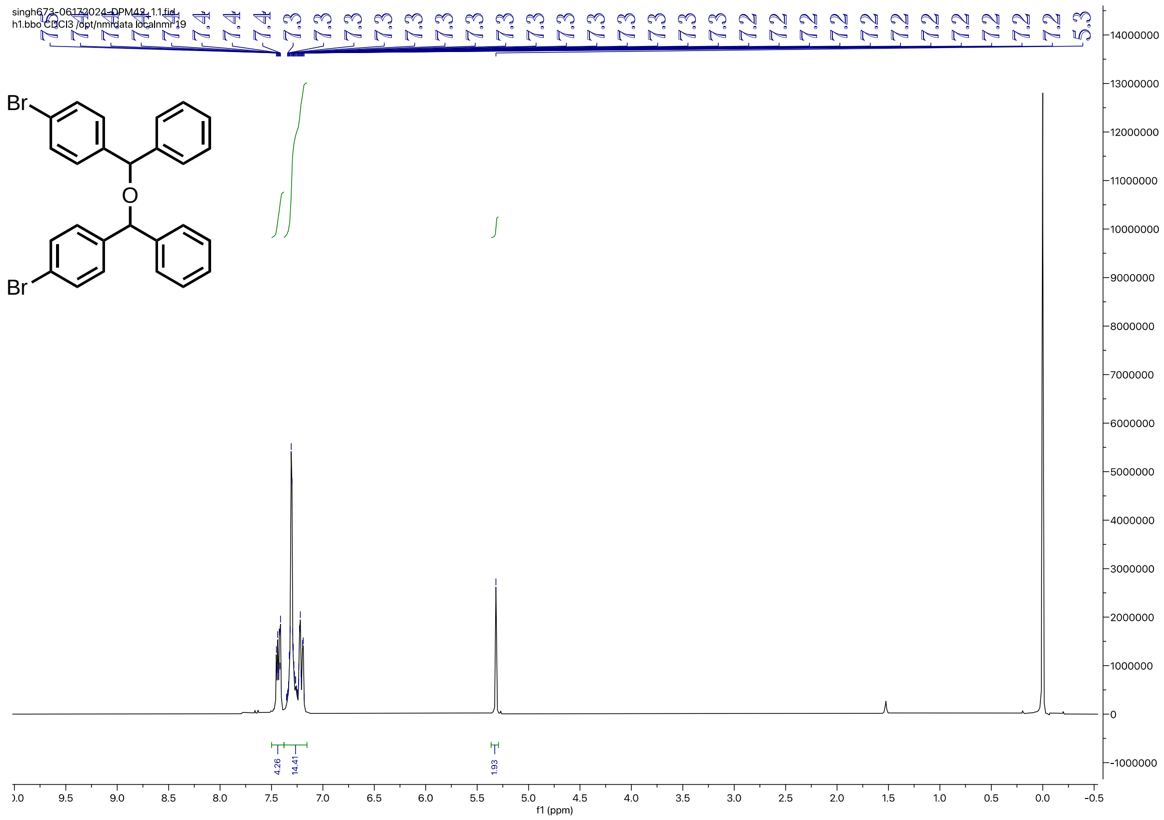

^{1}H NMR (300 MHz, Chloroform-*d*) 4,4'-(oxybis(phenylmethylene))bis(bromobenzene) **(2b)**

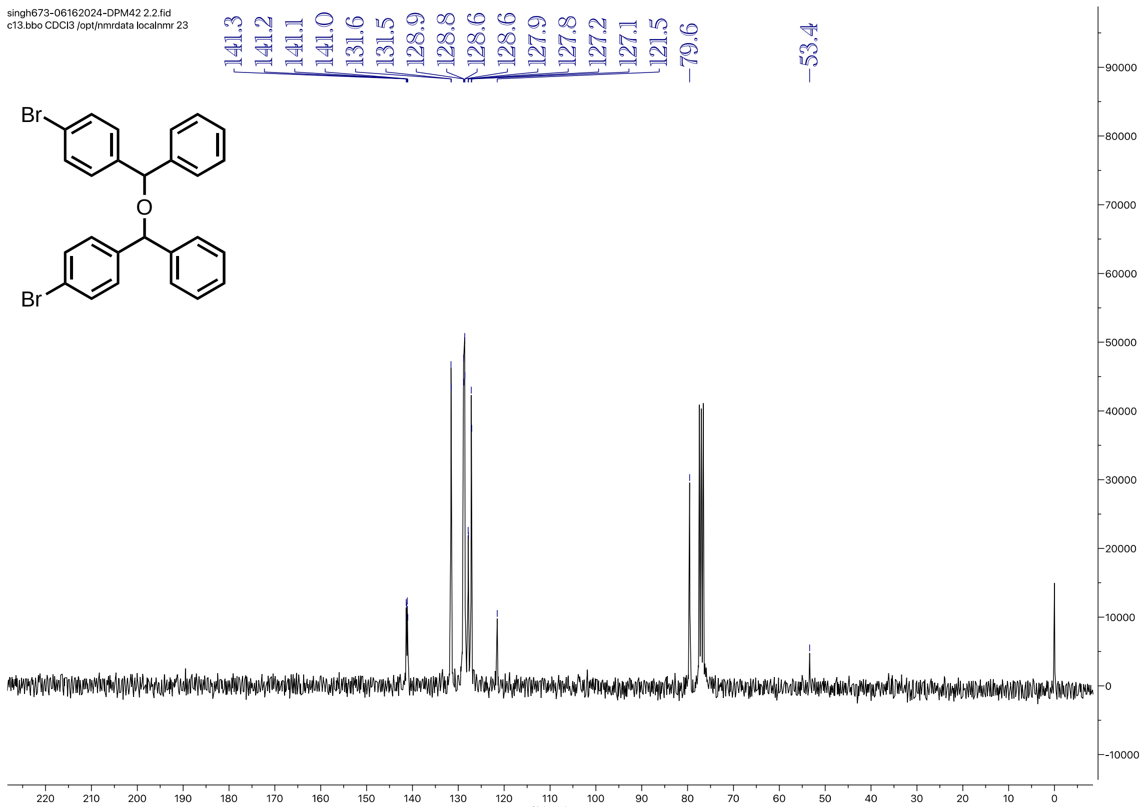

^{13}C {^{1}H} NMR (75 MHz, Chloroform-*d*) 4,4'-(oxybis(phenylmethylene))bis(bromobenzene) **(2b)**

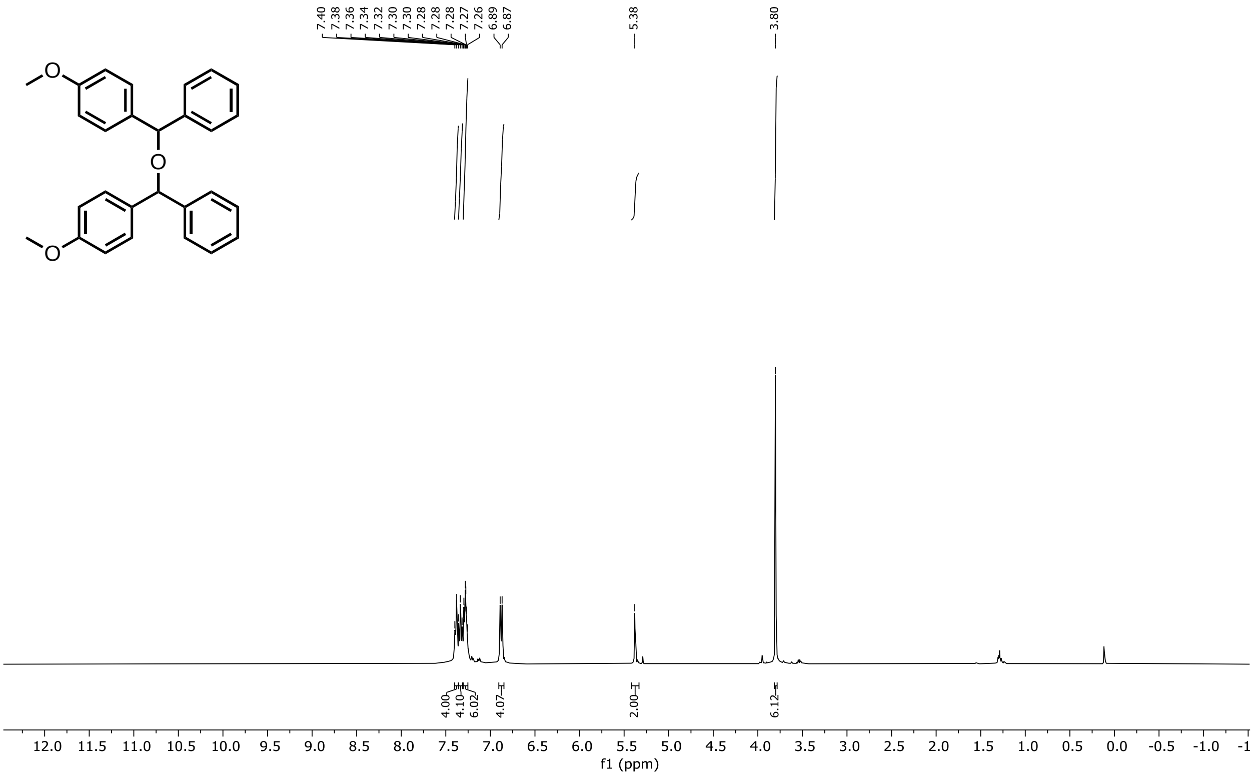

^{1}H NMR (400 MHz, Chloroform-*d*) 4,4'-(oxybis(phenylmethylene))bis(methoxybenzene) **(2c)**

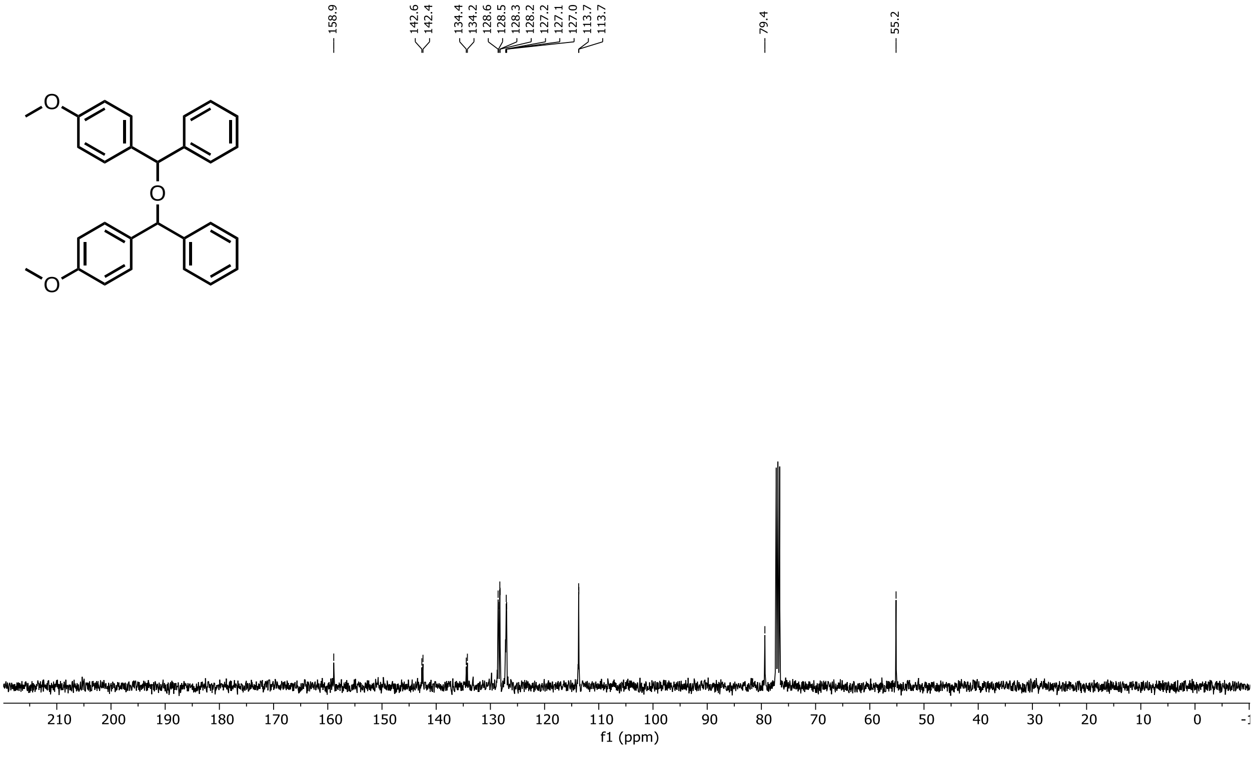

^{13}C {^{1}H} NMR (101 MHz, Chloroform-*d*) 4,4'-(oxybis(phenylmethylene))bis(methoxybenzene) **(2c)**

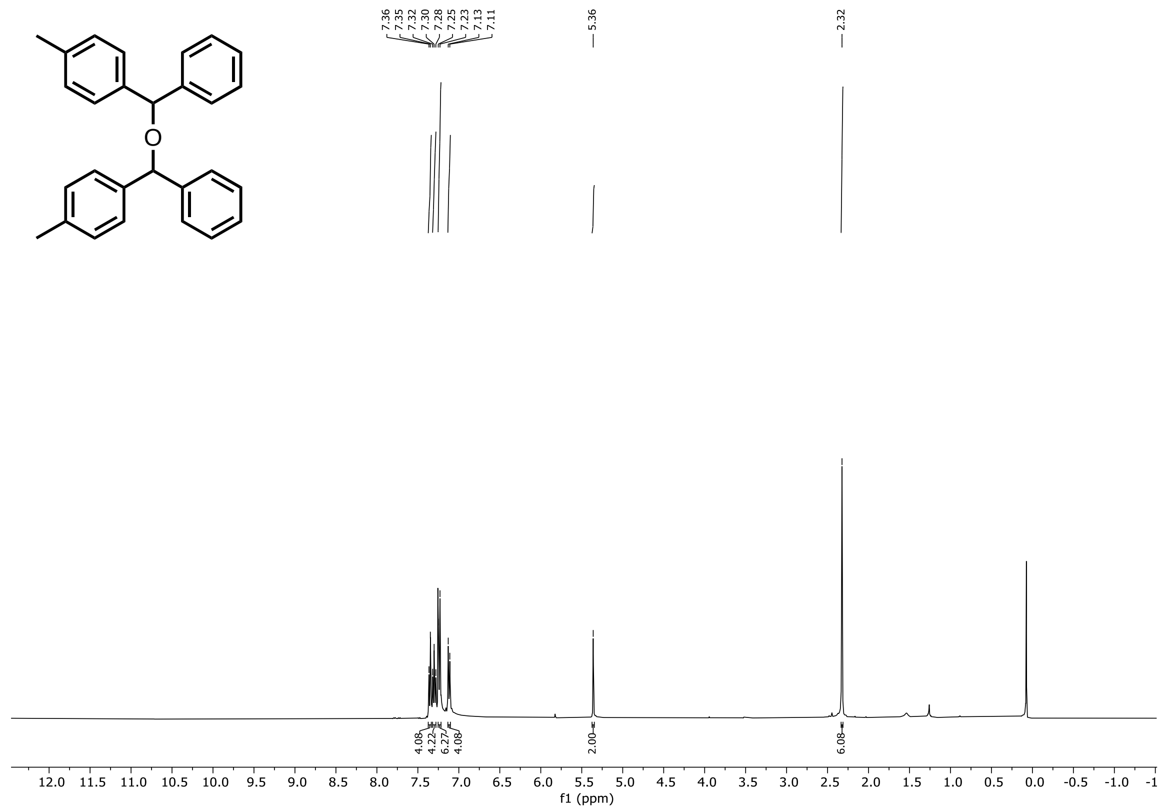

^{1}H NMR (400 MHz, Chloroform-*d*) 4,4'-(oxybis(phenylmethylene))bis(methylbenzene) **(2d)**

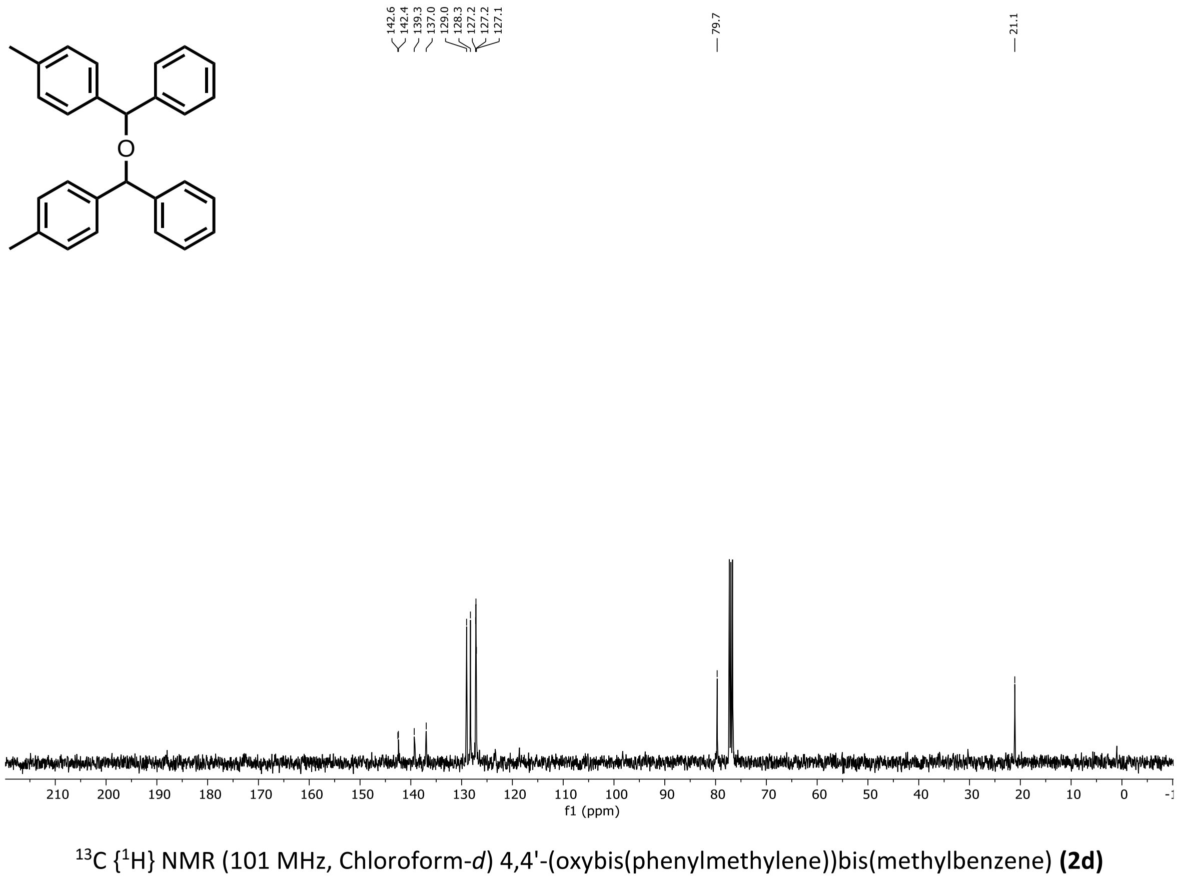

^{13}C {^{1}H} NMR (101 MHz, Chloroform-*d*) 4,4'-(oxybis(phenylmethylene))bis(methylbenzene) **(2d)**

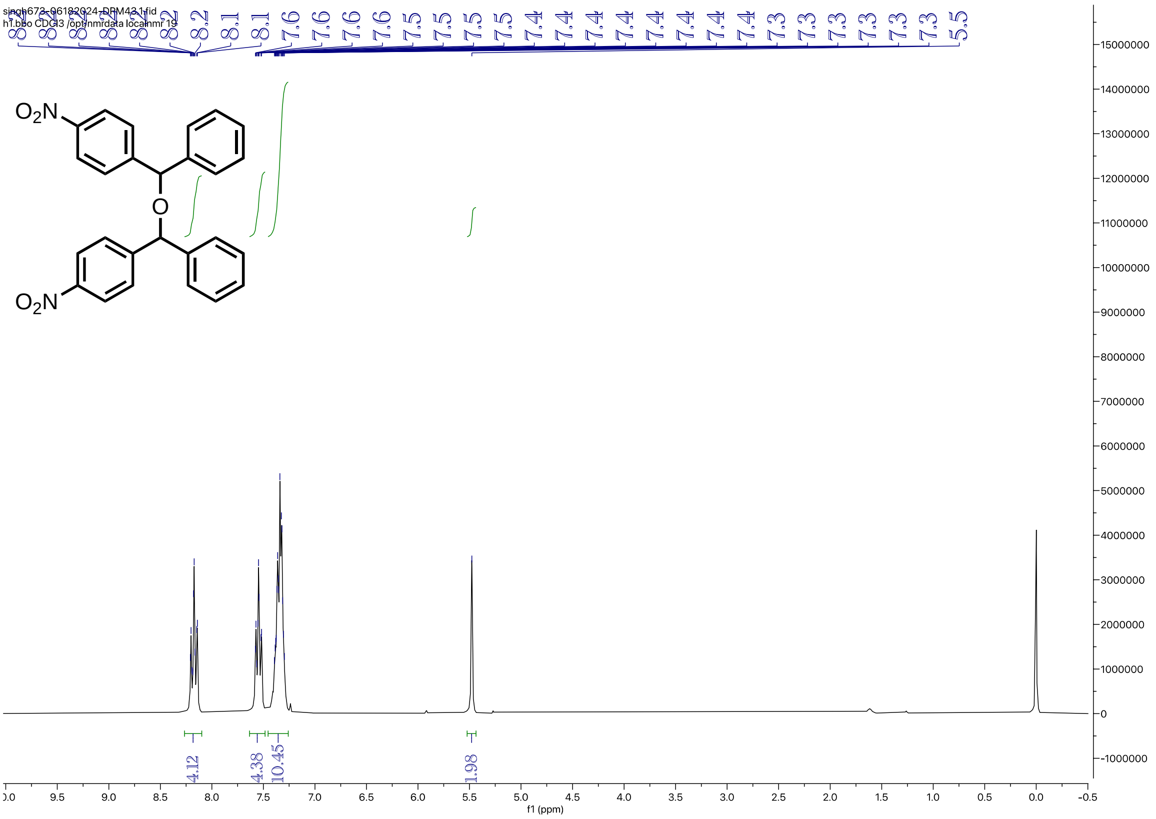

^1H NMR (300 MHz, Chloroform-*d*) 4,4'-(oxybis(phenylmethylene))bis(nitrobenzene) **(2e)**

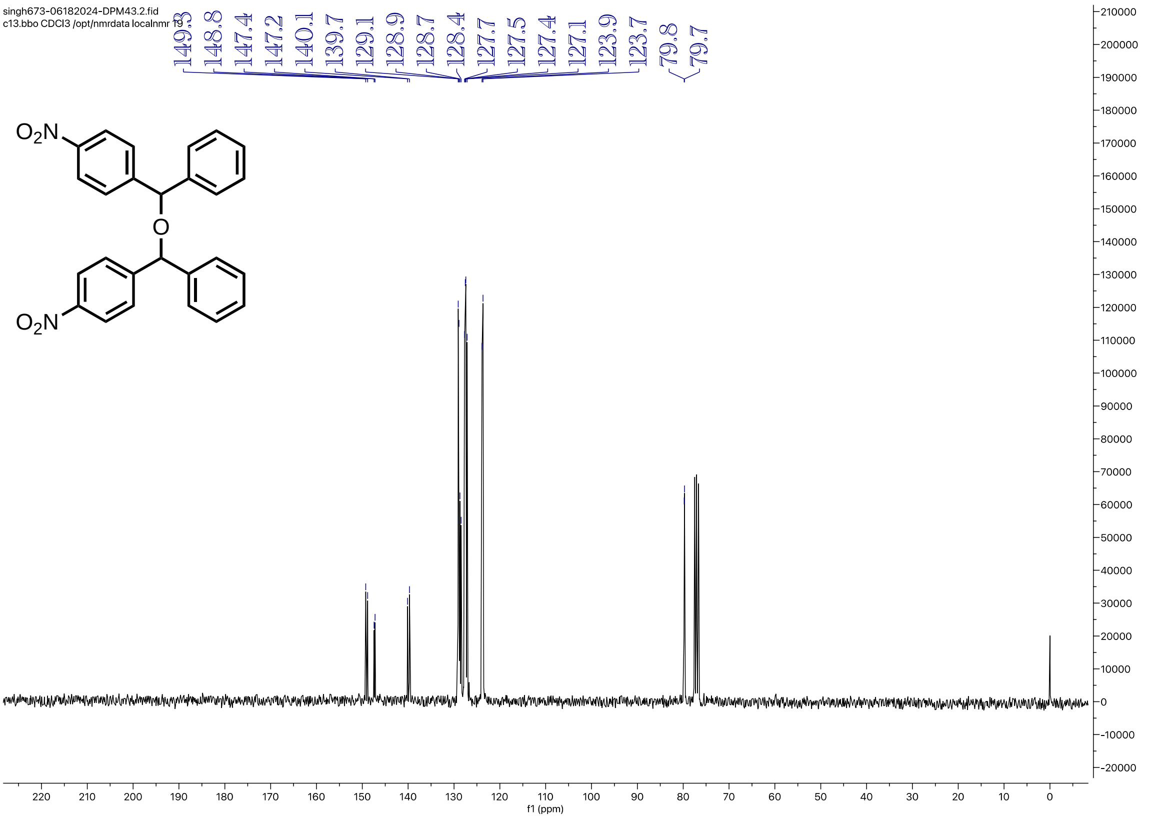

^{13}C {^1H} NMR (75 MHz, Chloroform-*d*) 4,4'-(oxybis(phenylmethylene))bis(nitrobenzene) **(2e)**

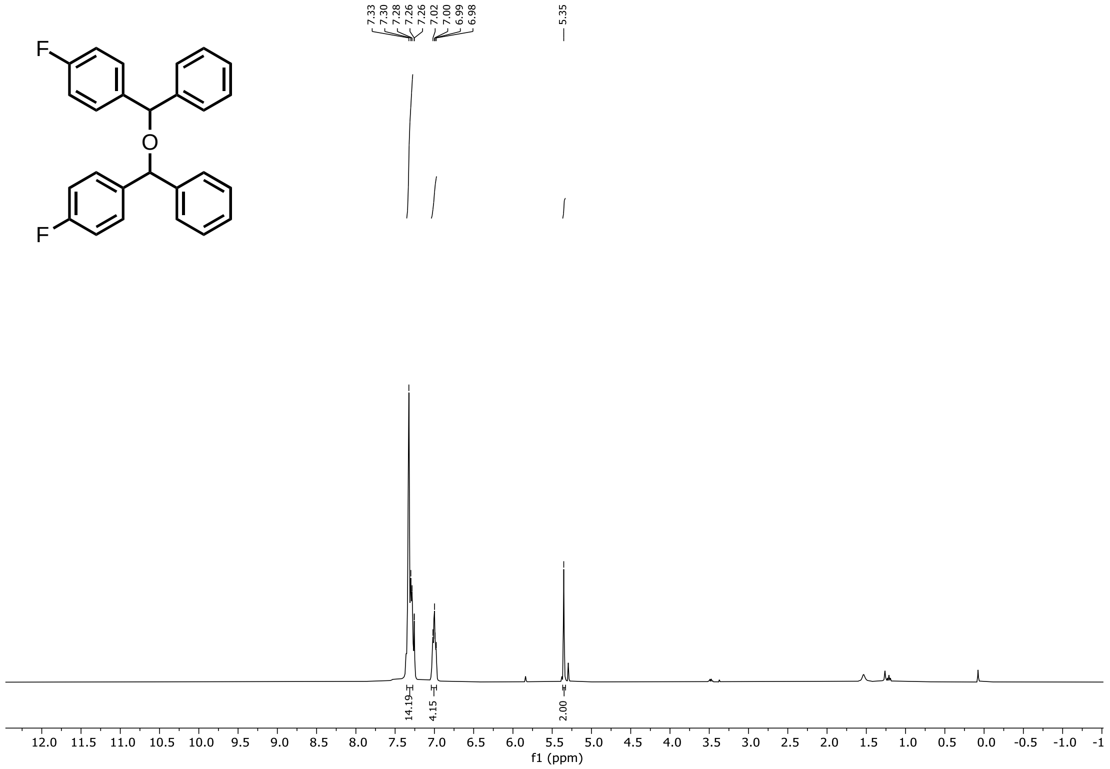

^{1}H NMR (400 MHz, Chloroform-*d*) 4,4'-(oxybis(phenylmethylene))bis(fluorobenzene) **(2f)**

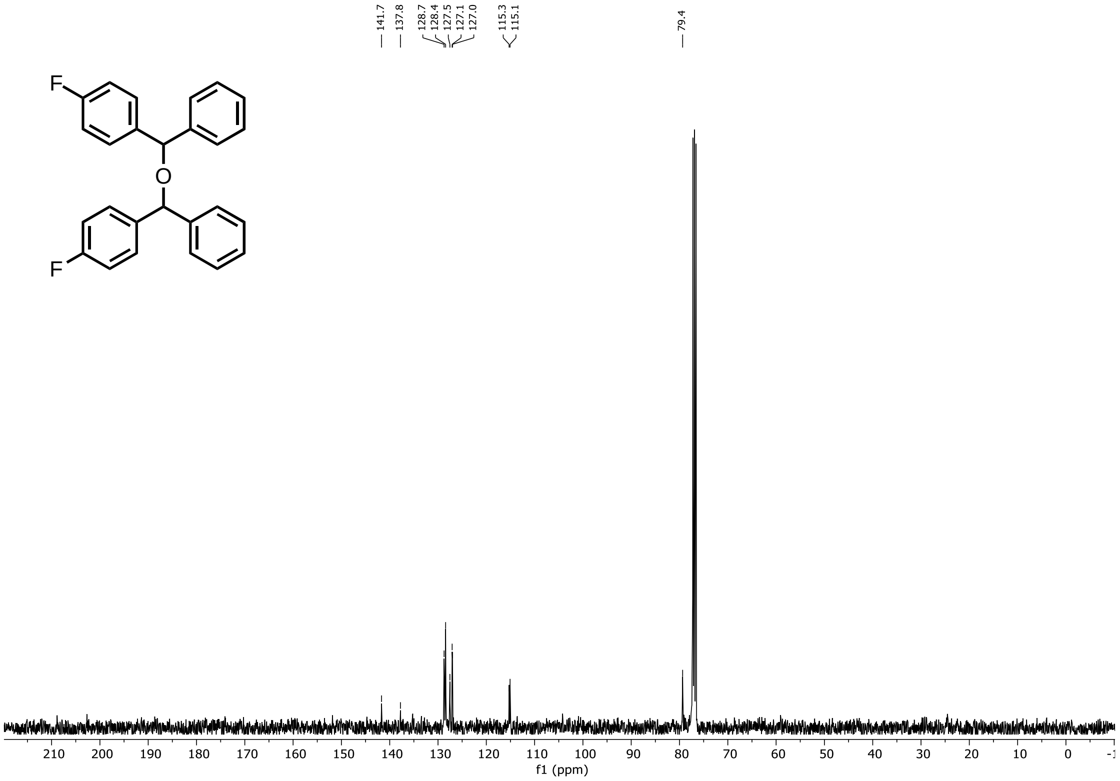

^{13}C {^{1}H} NMR (101 MHz, Chloroform-*d*) 4,4'-(oxybis(phenylmethylene))bis(fluorobenzene) **(2f)**

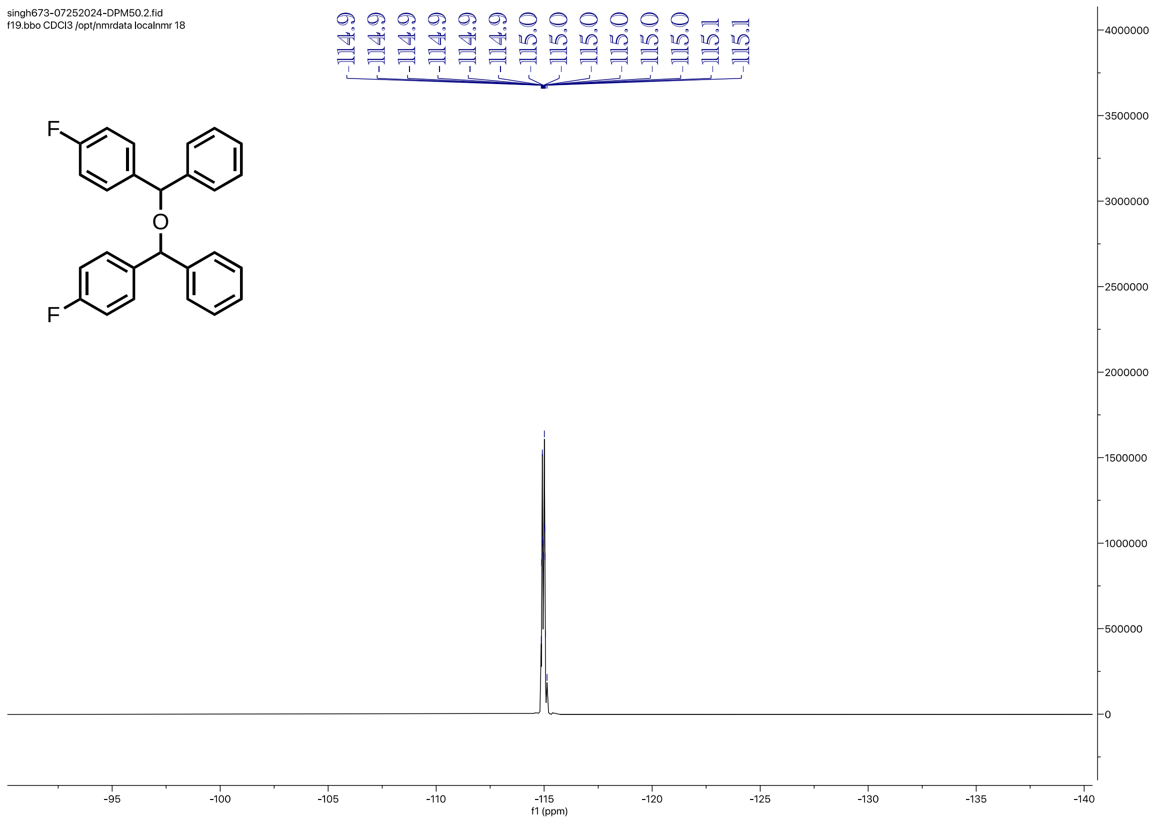

^{19}F NMR (282 MHz, Chloroform-*d*) 4,4'-(oxybis(phenylmethylene))bis(fluorobenzene) **(2f)**

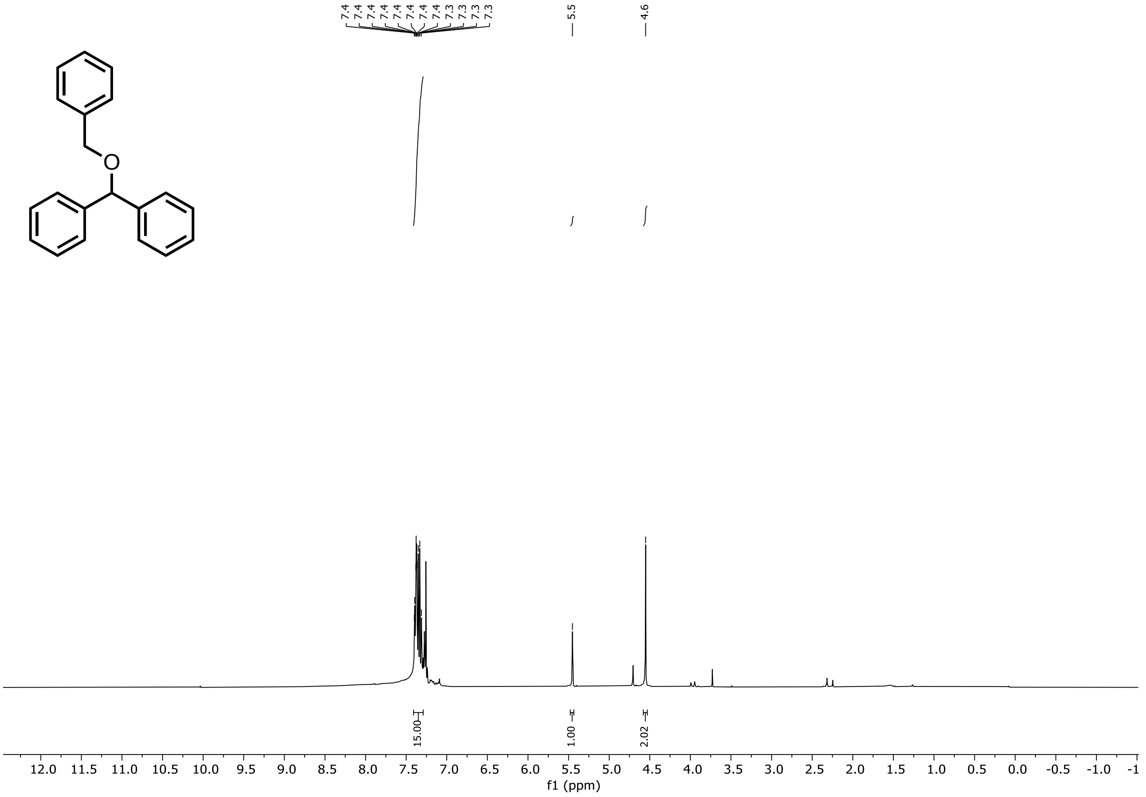

^{1}H NMR (400 MHz, Chloroform-*d*) ((benzyloxy)methylene)dibenzene **(4aa)**

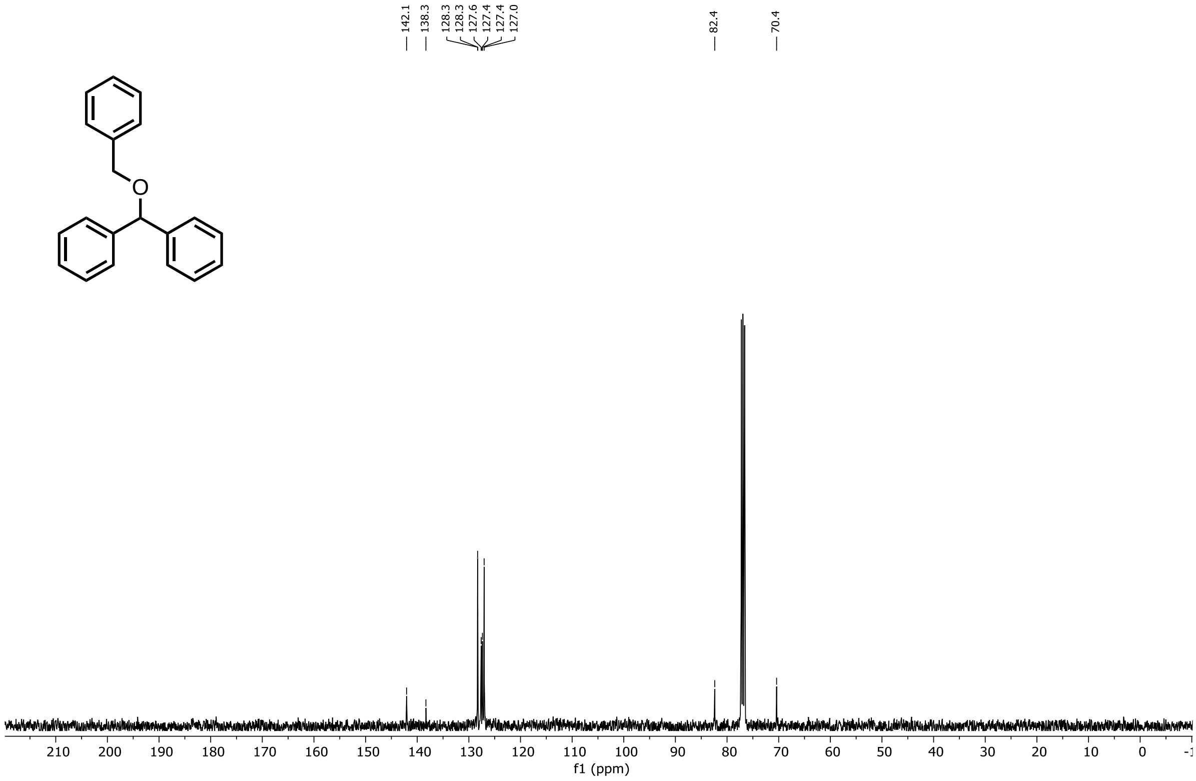

^{13}C {^{1}H} NMR (101 MHz, Chloroform-*d*) ((benzyloxy)methylene)dibenzene **(4aa)**

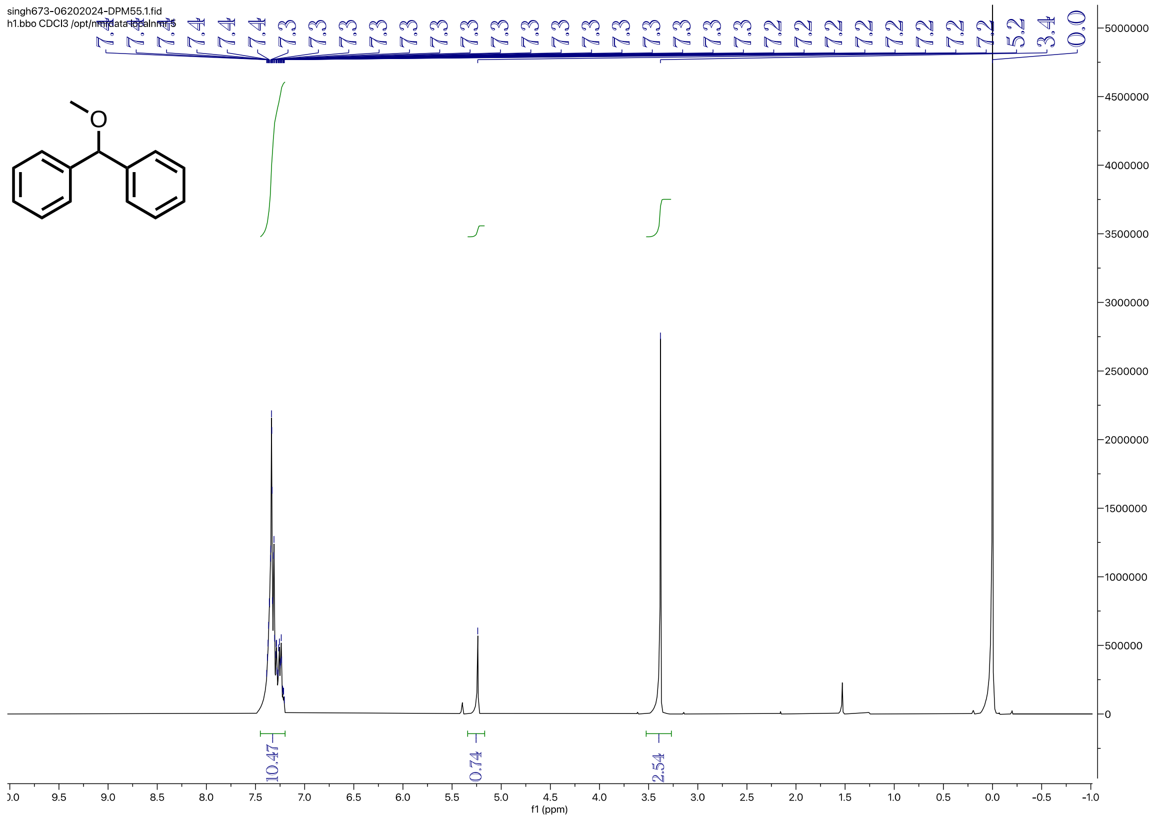

^{1}H NMR (300 MHz, Chloroform-*d*) (methoxymethylene)dibenzene **(4ab)**

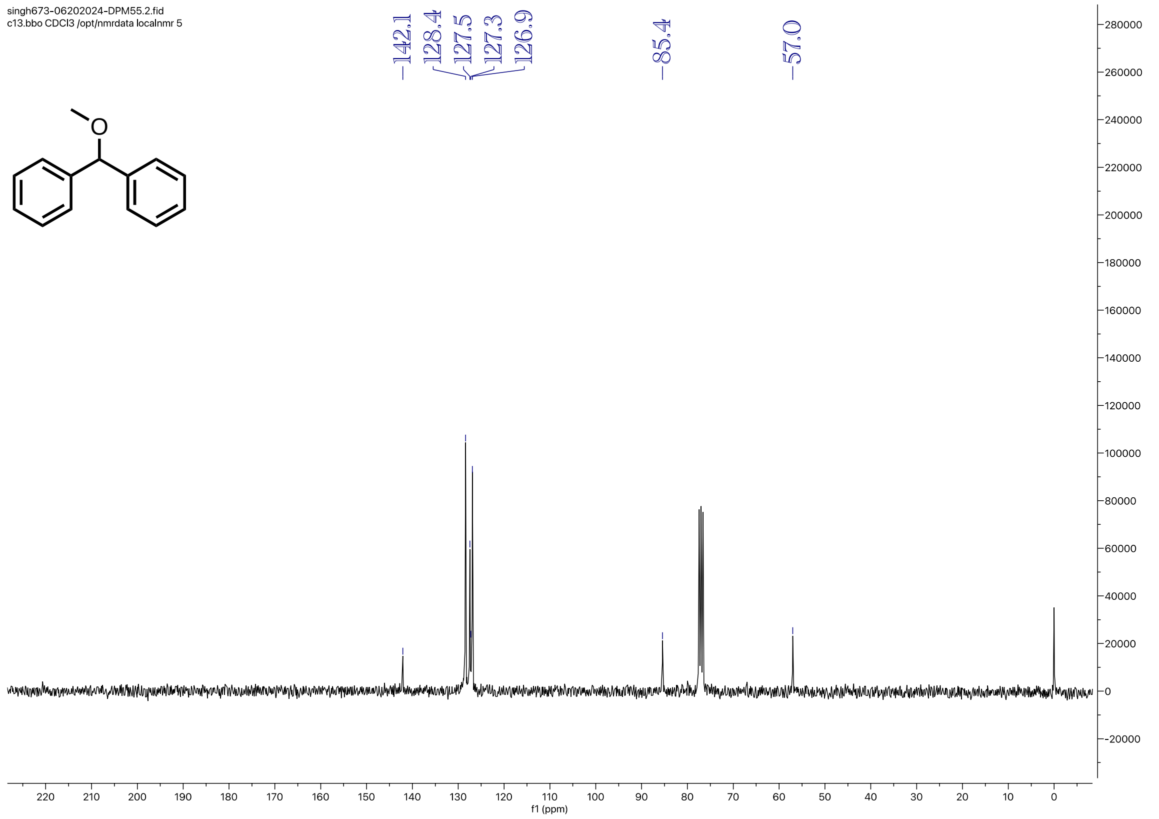

^{13}C {^{1}H} NMR (75 MHz, Chloroform-*d*) (methoxymethylene)dibenzene **(4ab)**

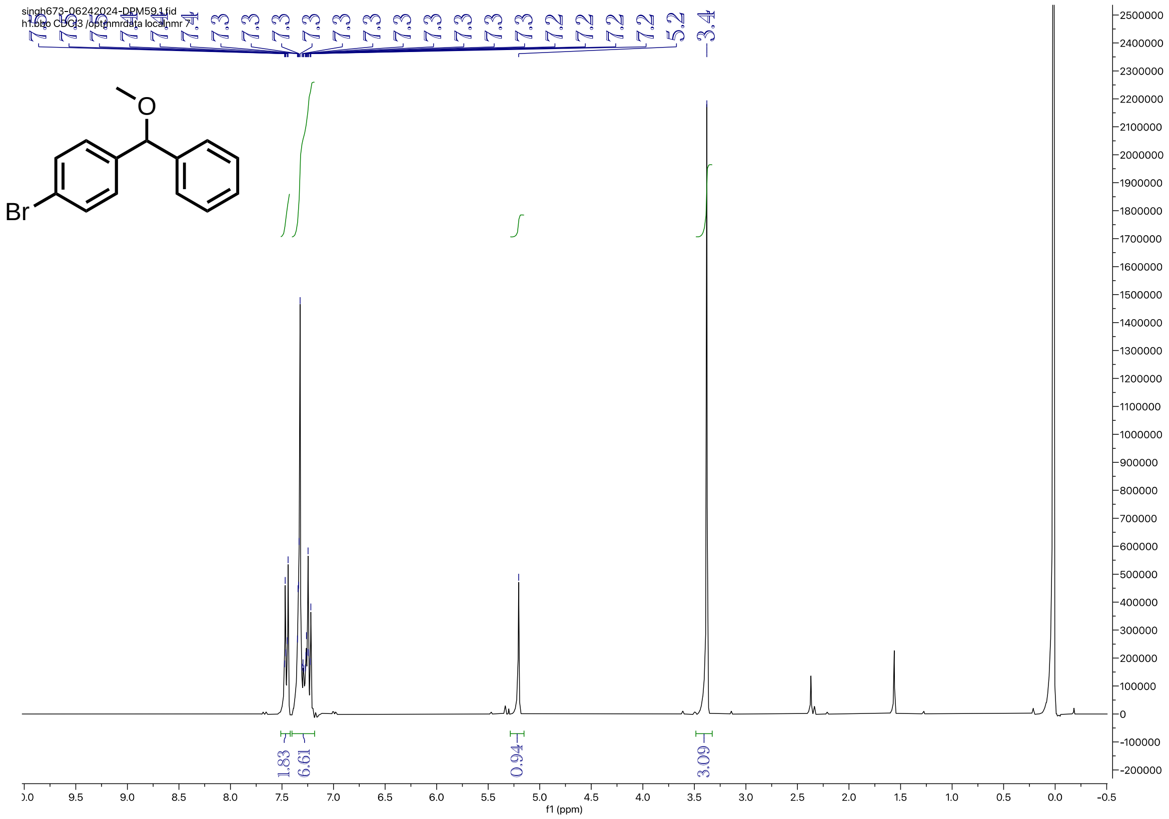

^{1}H NMR (300 MHz, Chloroform-*d*) 1-bromo-4-(methoxy(phenyl)methyl)benzene **(4bb)**

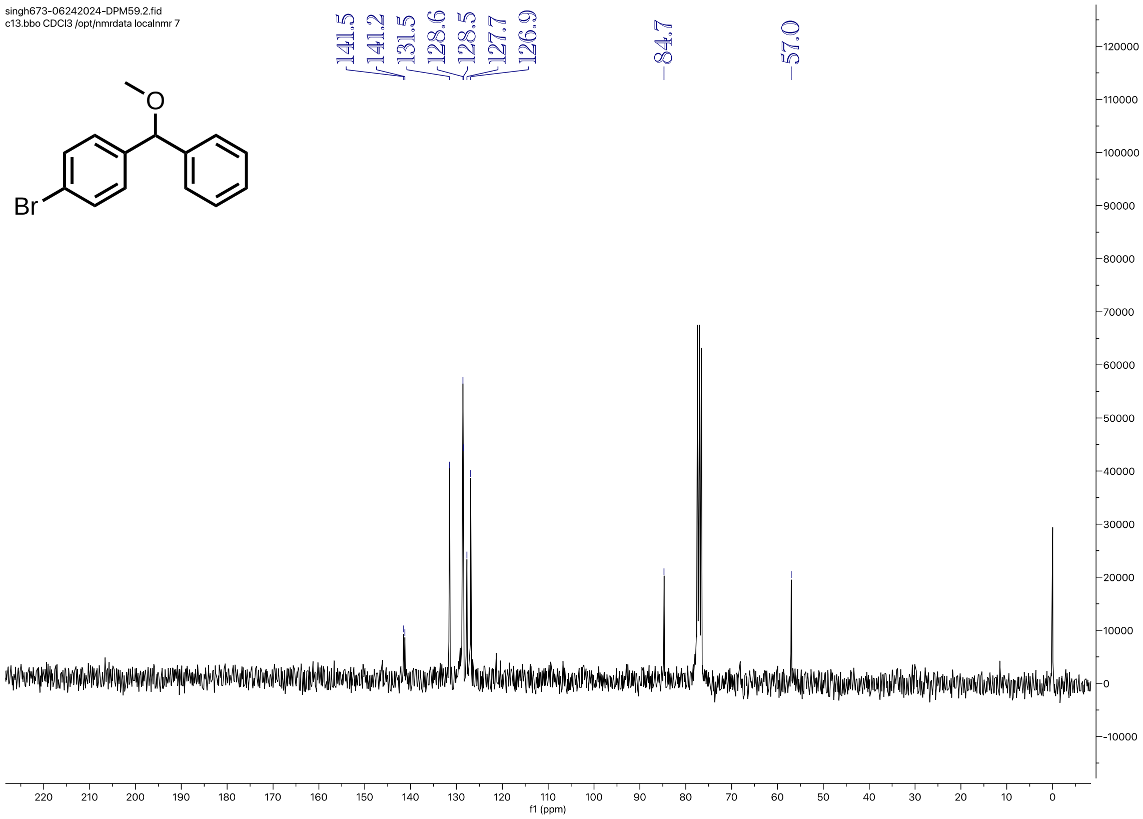

^{13}C {^{1}H} NMR (75 MHz, Chloroform-*d*) 1-bromo-4-(methoxy(phenyl)methyl)benzene **(4bb)**

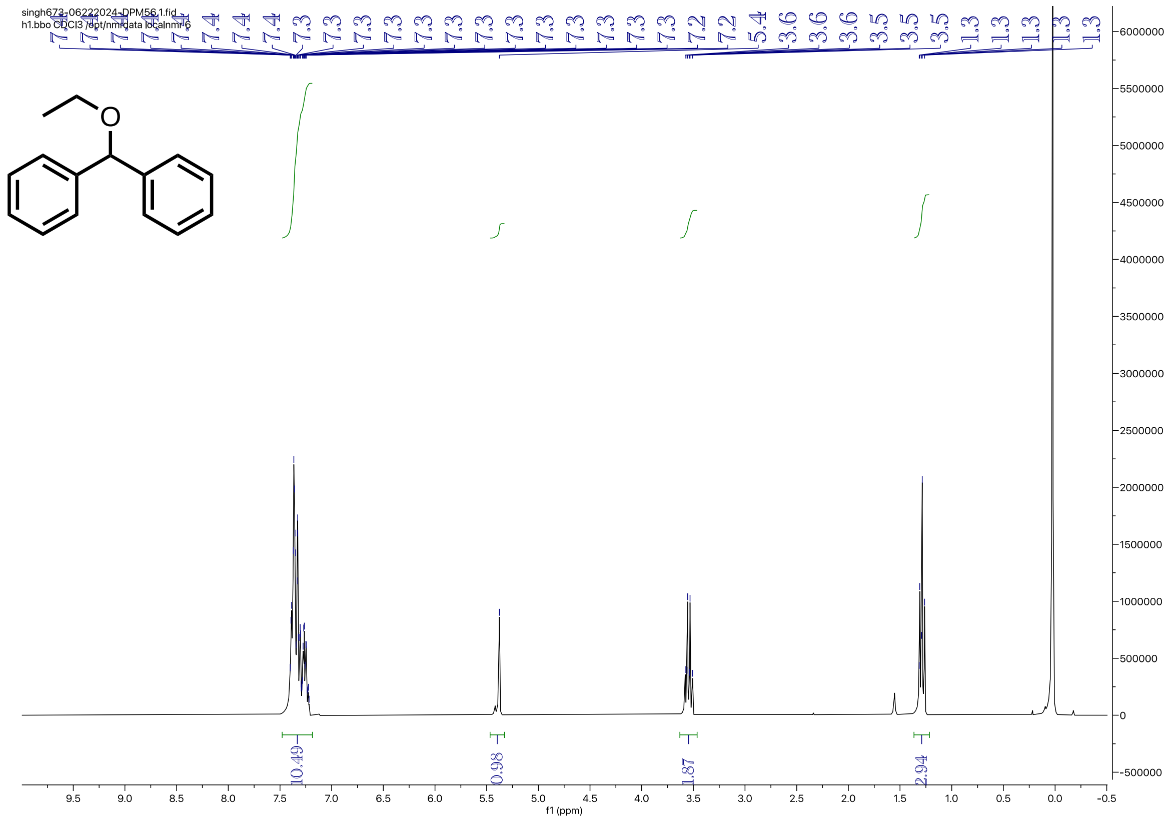

^{1}H NMR (300 MHz, Chloroform-*d*) (ethoxymethylene)dibenzene **(4ac)**

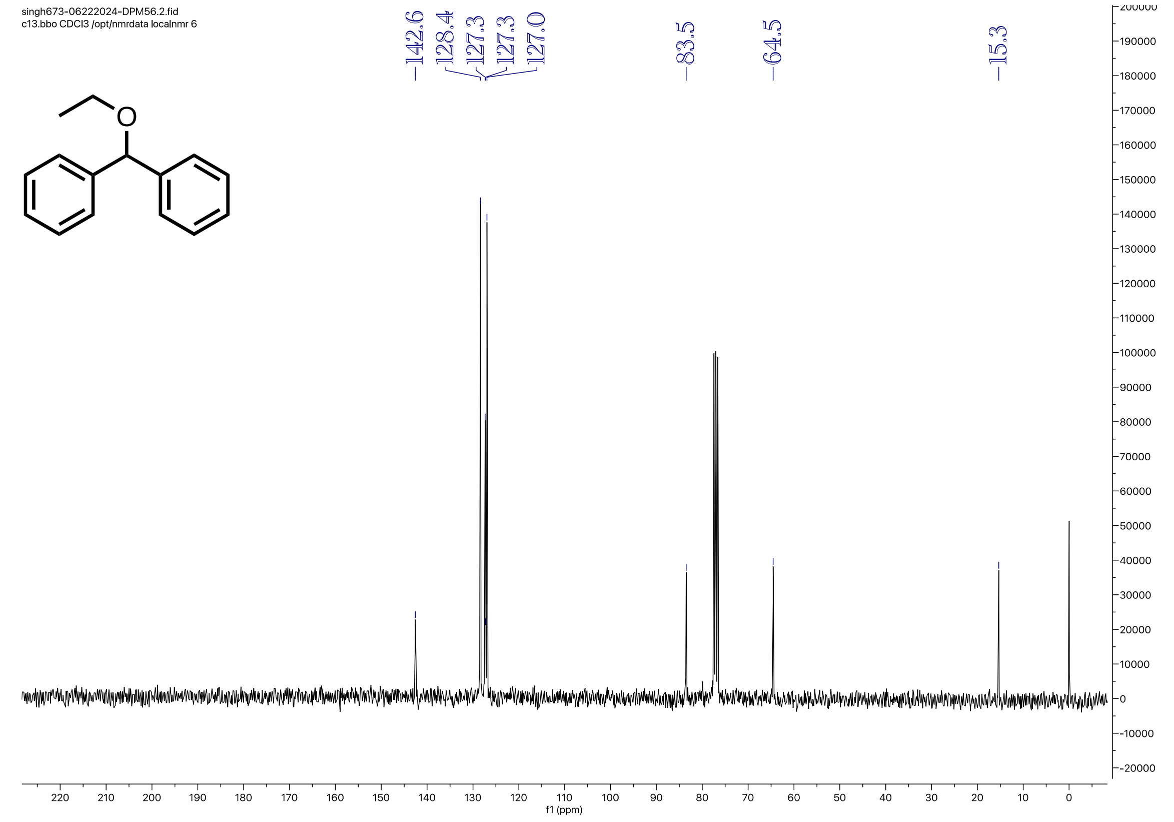

^{13}C {^{1}H} NMR (75 MHz, Chloroform-*d*) **(**ethoxymethylene)dibenzene **(4ac)**

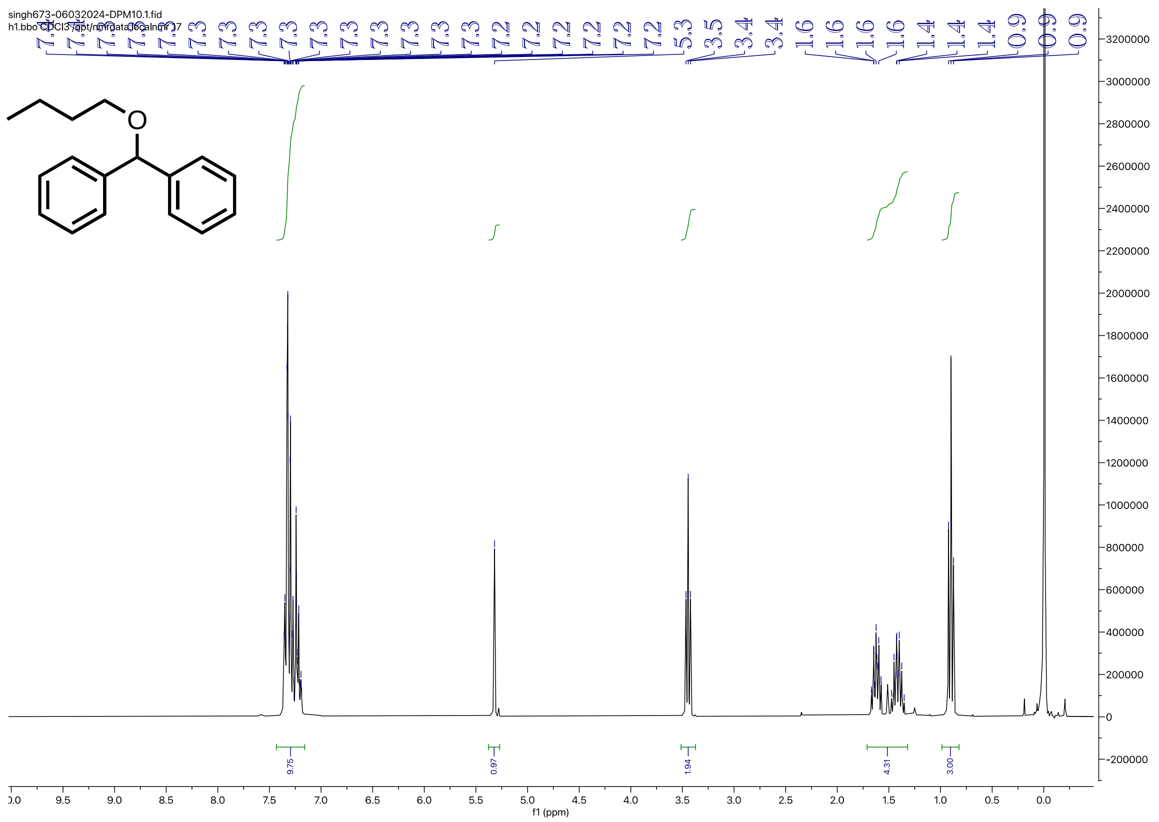

^{1}H NMR (300 MHz, Chloroform-*d*) (butoxymethylene)dibenzene **(4ad)**

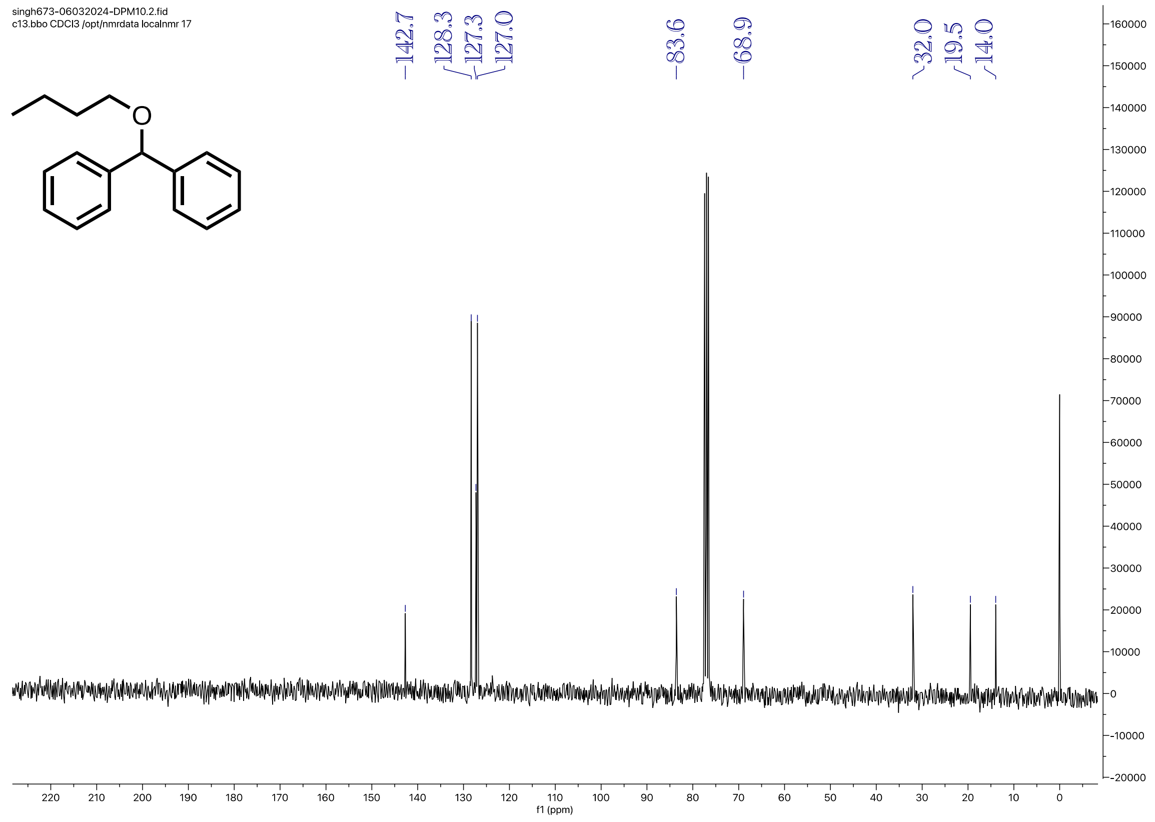

^{13}C {^{1}H} NMR (75 MHz, Chloroform-*d*) (butoxymethylene)dibenzene **(4ad)**

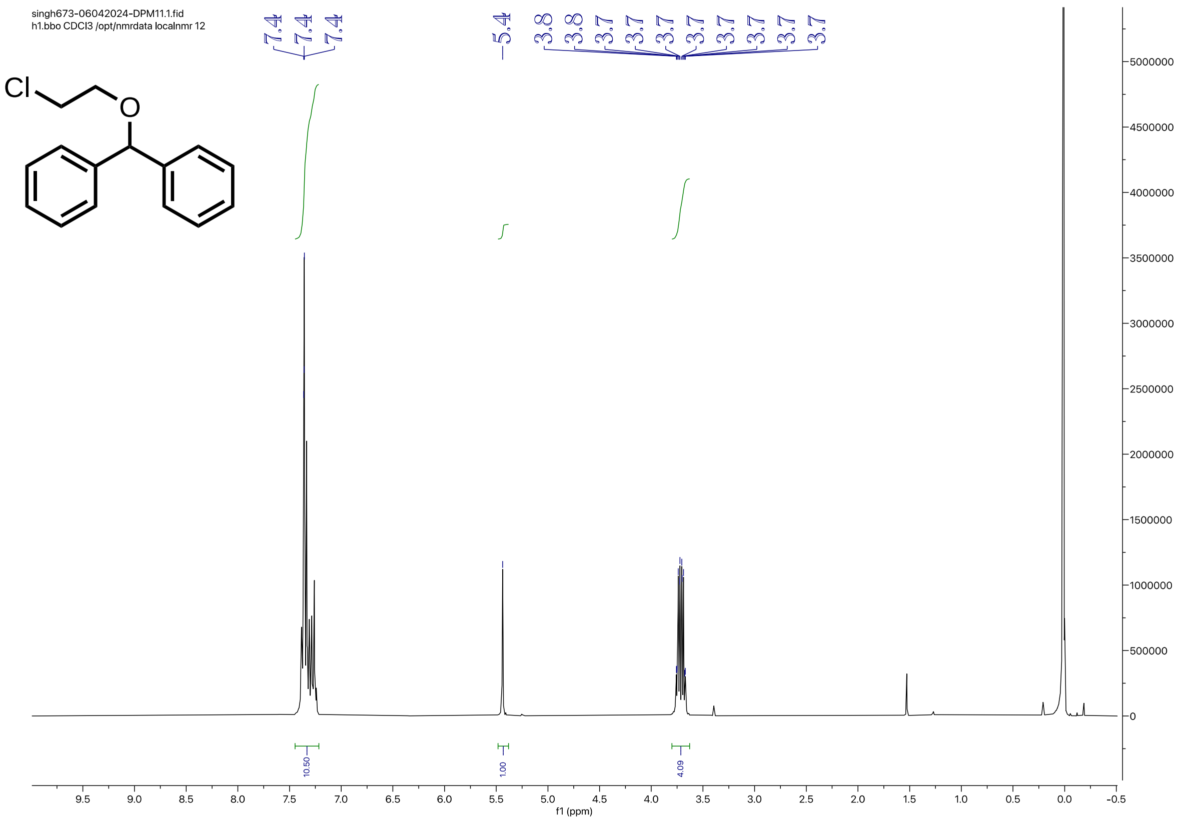

^{1}H NMR (300 MHz, Chloroform-*d*) ((2-chloroethoxy)methylene)dibenzene **(4ae)**

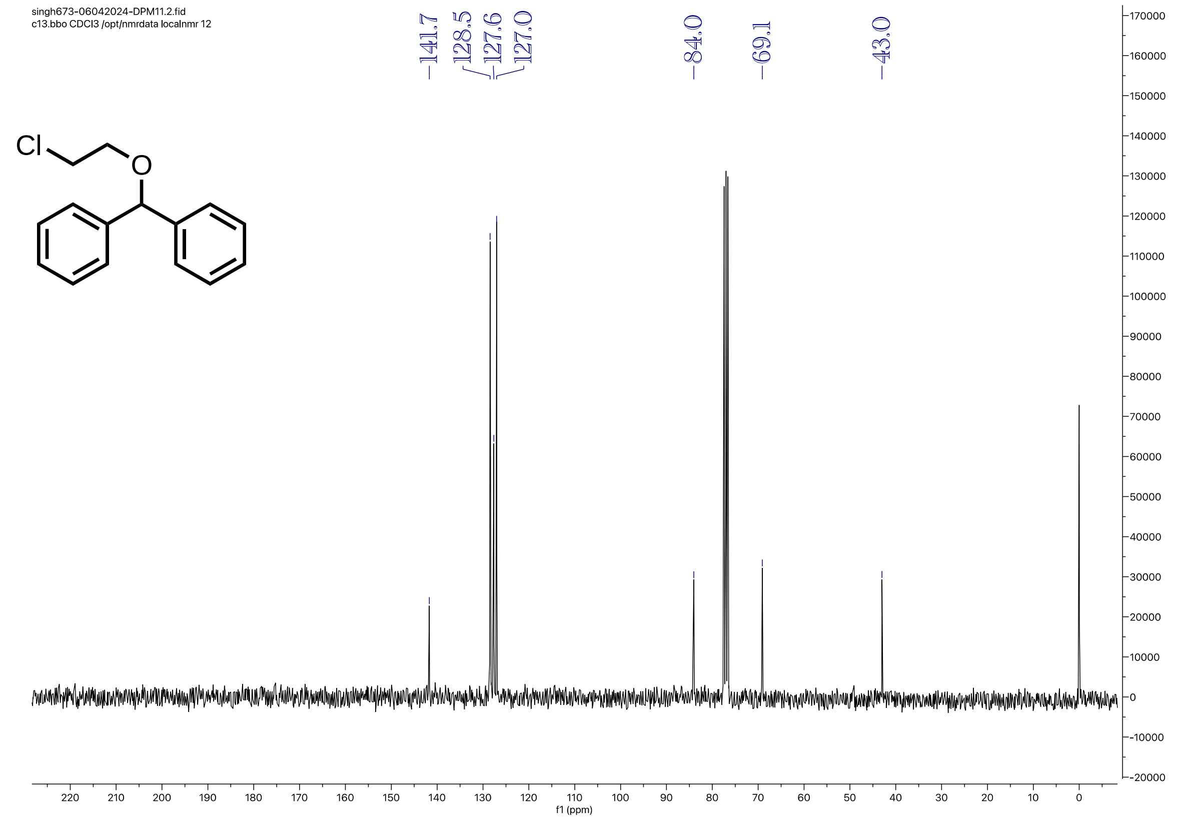

^{13}C {^{1}H} NMR (75 MHz, Chloroform-*d*) ((2-chloroethoxy)methylene)dibenzene **(4ae)**

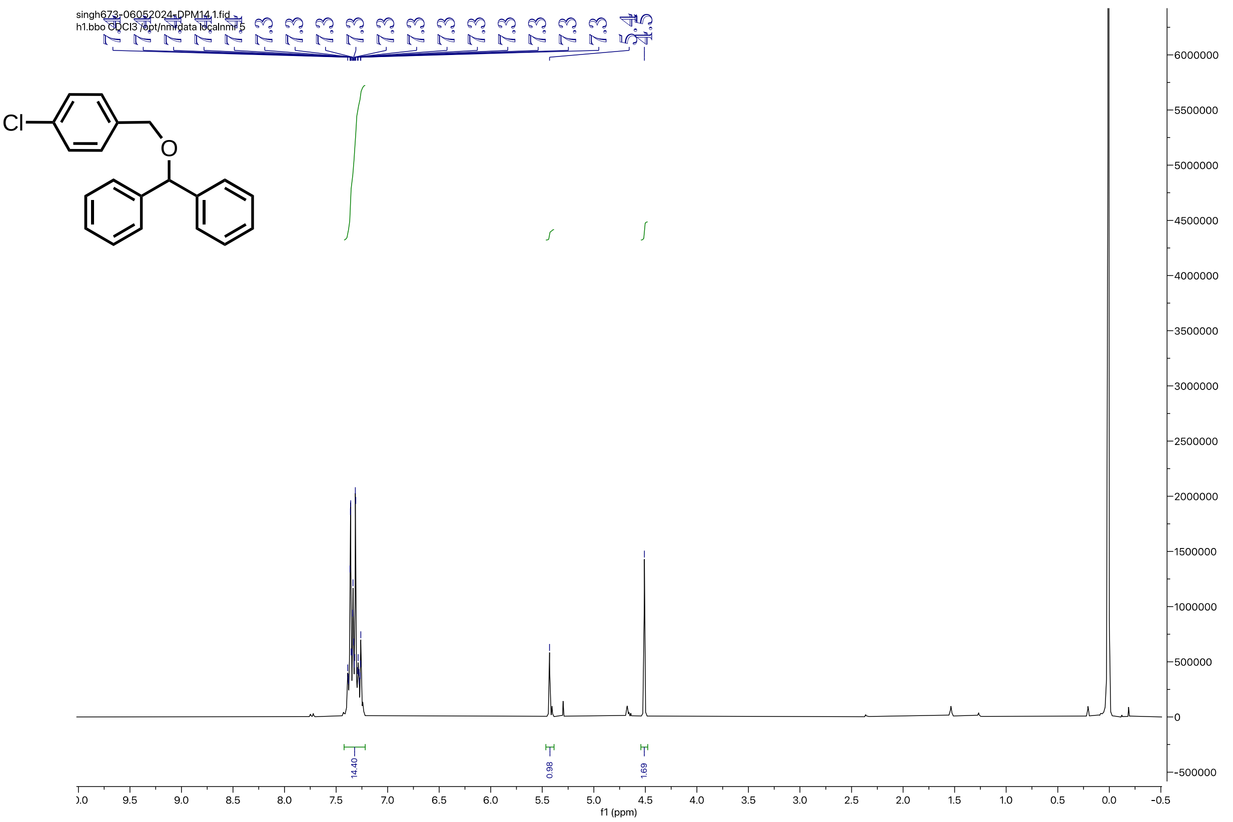

^{1}H NMR (300 MHz, Chloroform-*d*) (((4-chlorobenzyl)oxy)methylene)dibenzene **(4af)**

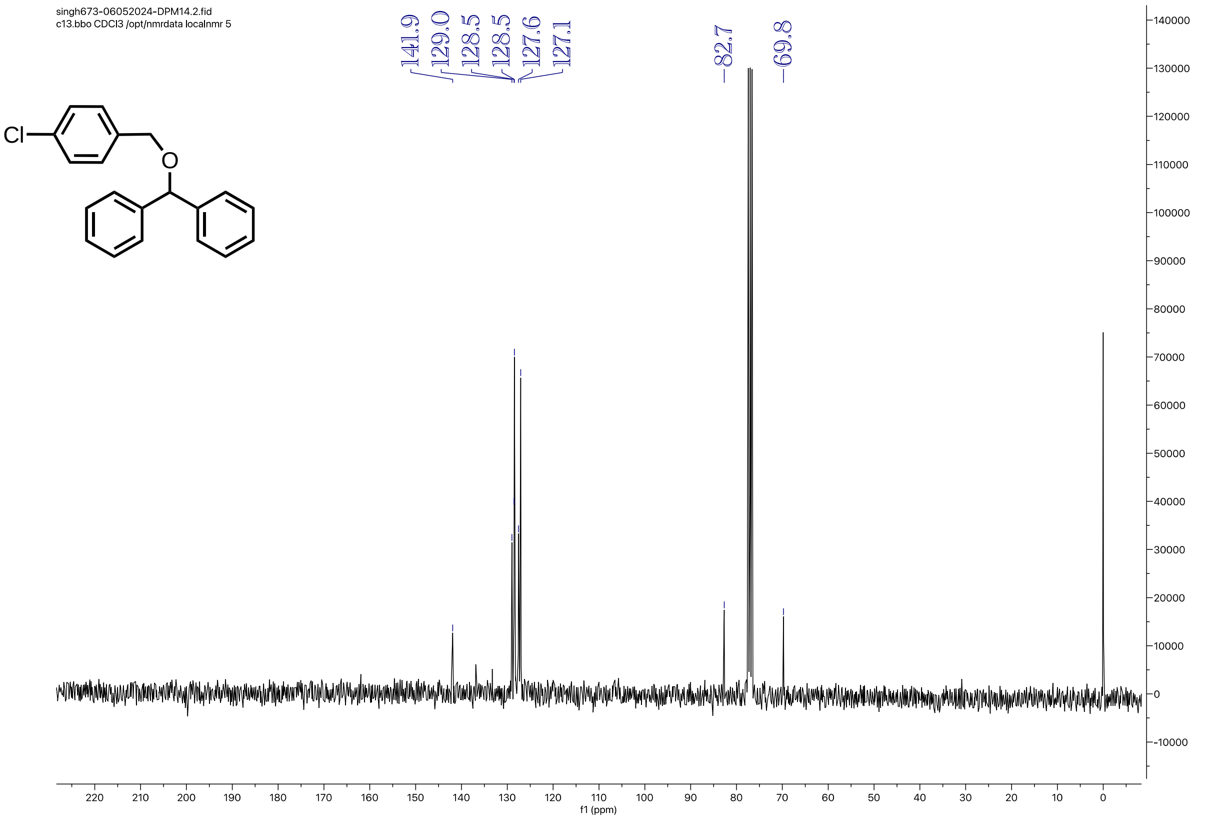

^{13}C {^{1}H} NMR (75 MHz, Chloroform-*d*) (((4-chlorobenzyl)oxy)methylene)dibenzene **(4af)**

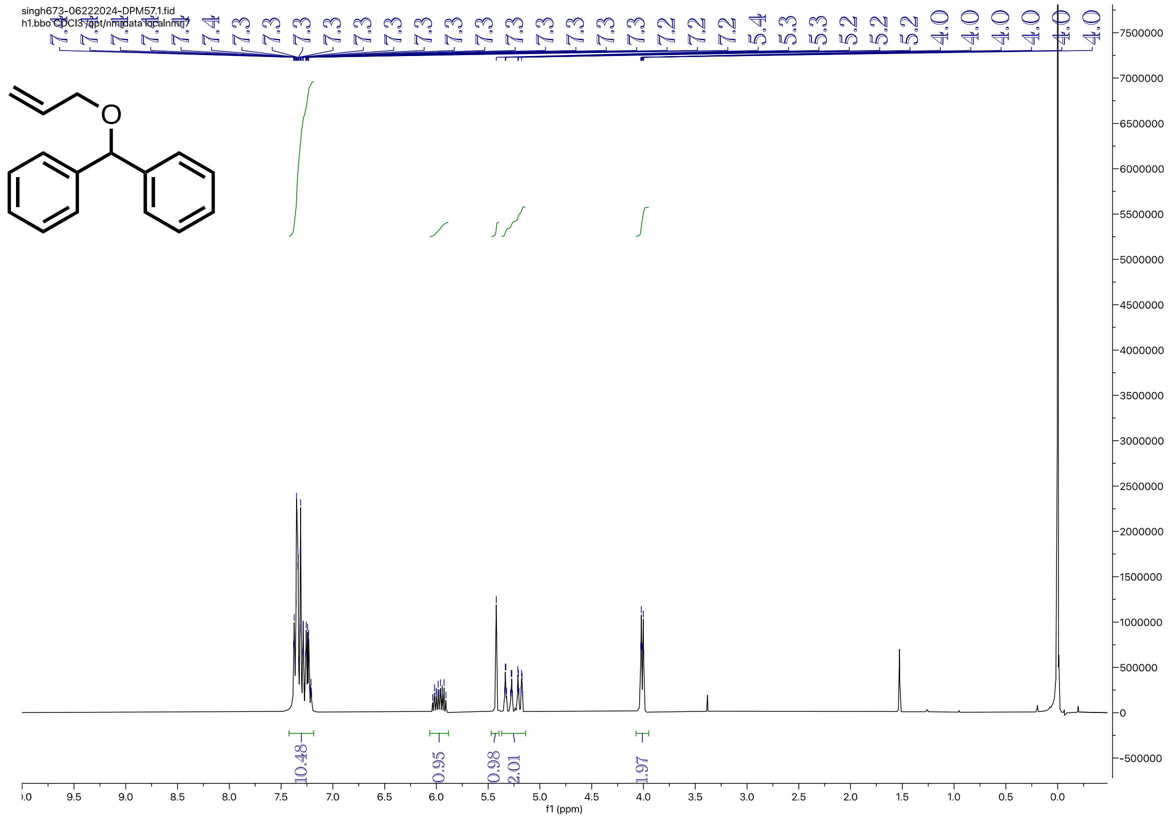

^{1}H NMR (300 MHz, Chloroform-*d*) ((allyloxy)methylene)dibenzene **(4ai)**

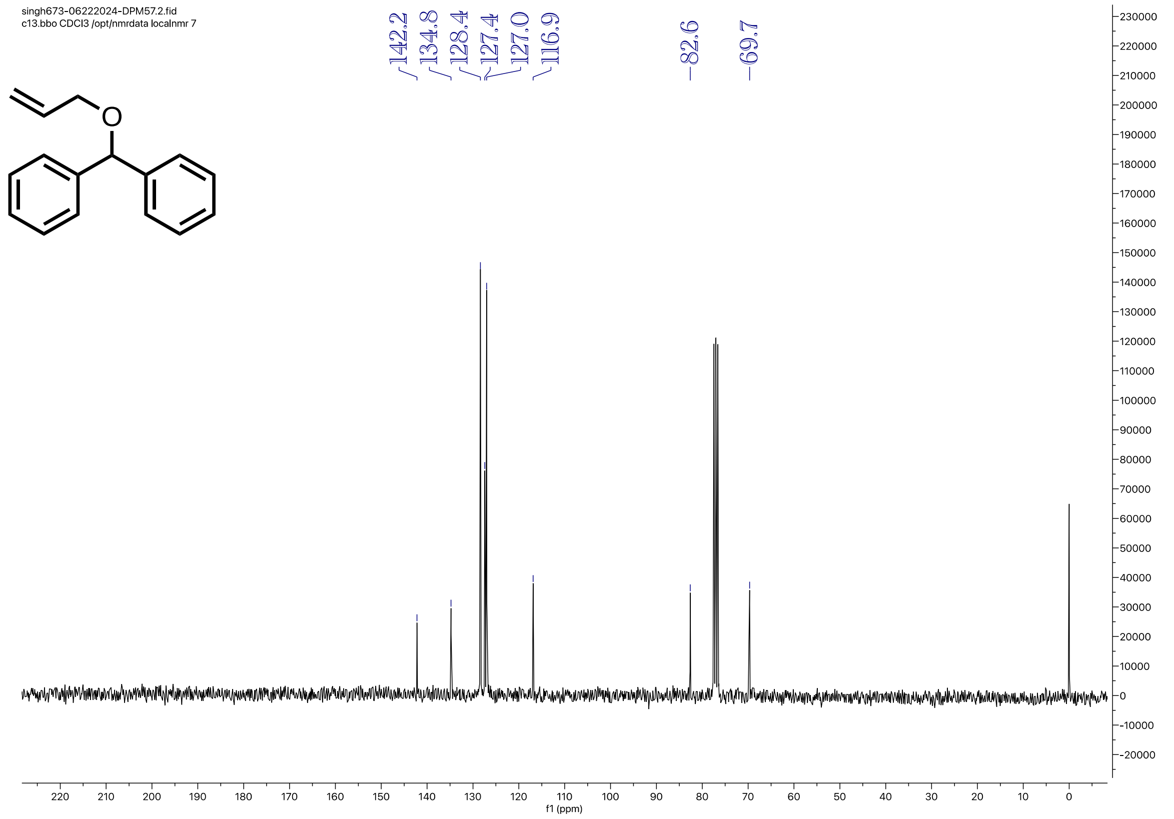

^{13}C {^{1}H} NMR (75 MHz, Chloroform-*d*) ((allyloxy)methylene)dibenzene **(4ai)**

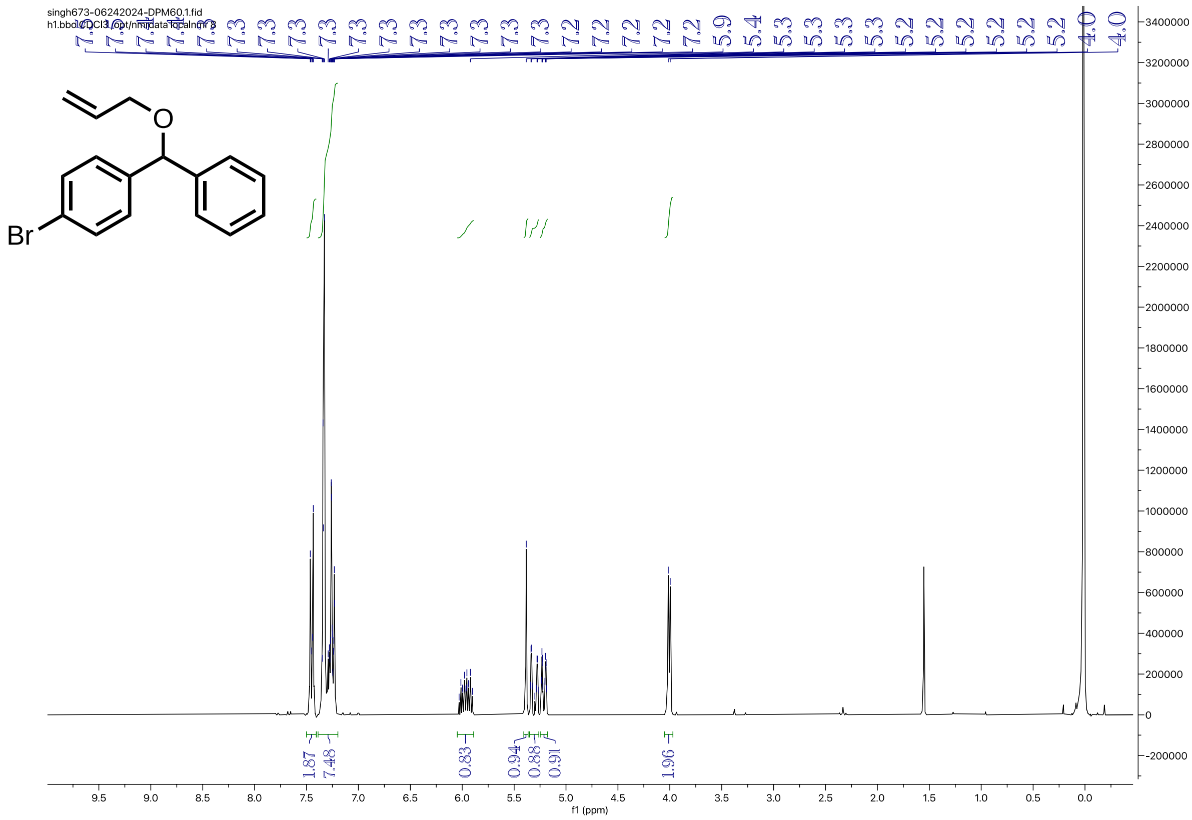

^{1}H NMR (300 MHz, Chloroform-*d*) 1-((allyloxy)(phenyl)methyl)-4-bromobenzene **(4bi)**

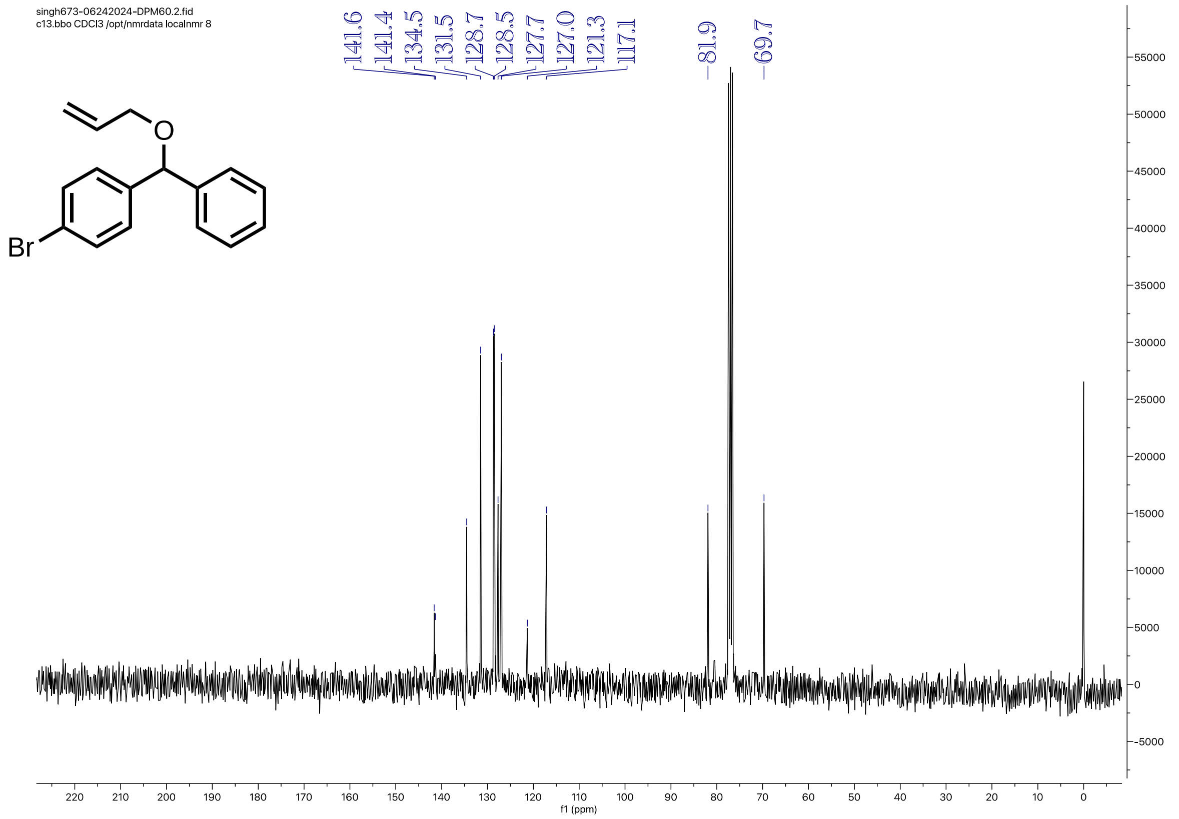

^{13}C {^{1}H} NMR (75 MHz, Chloroform-*d*) 1-((allyloxy)(phenyl)methyl)-4-bromobenzene **(4bi)**

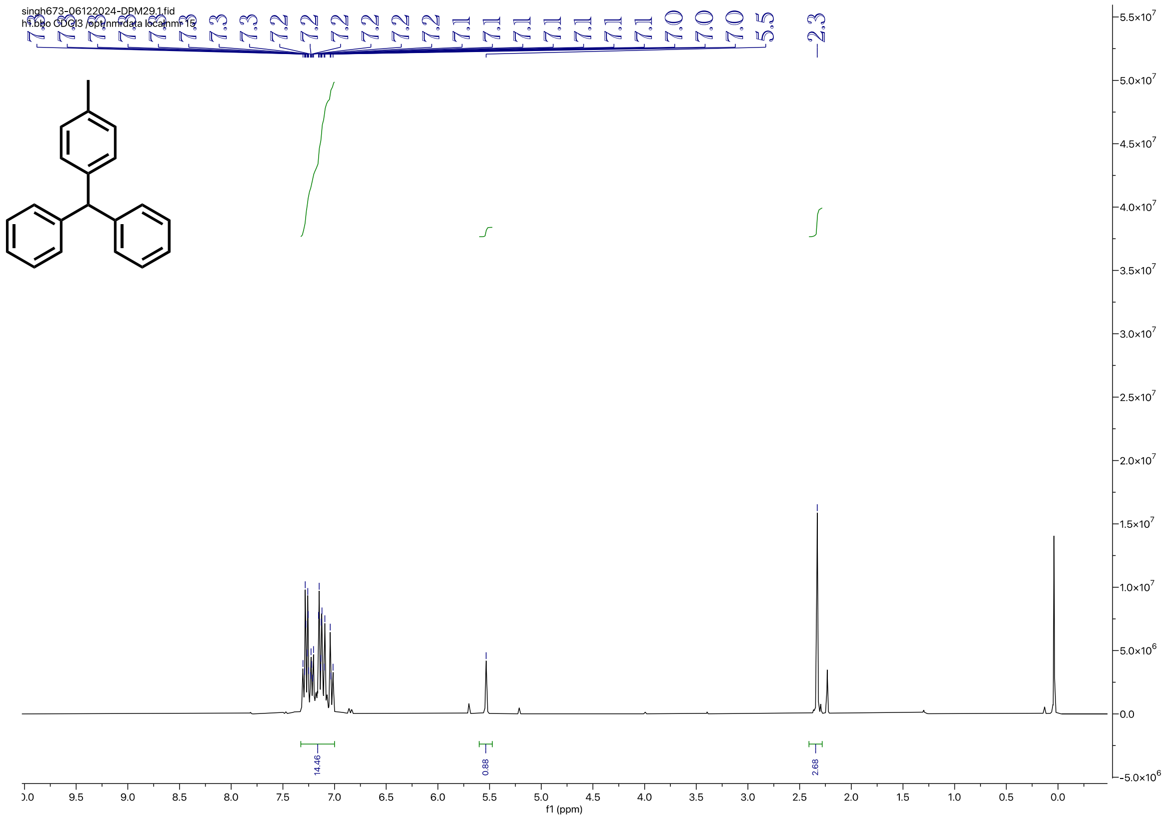

^{1}H NMR (300 MHz, Chloroform-*d*) (*p*-tolylmethylene)dibenzene **(5)**

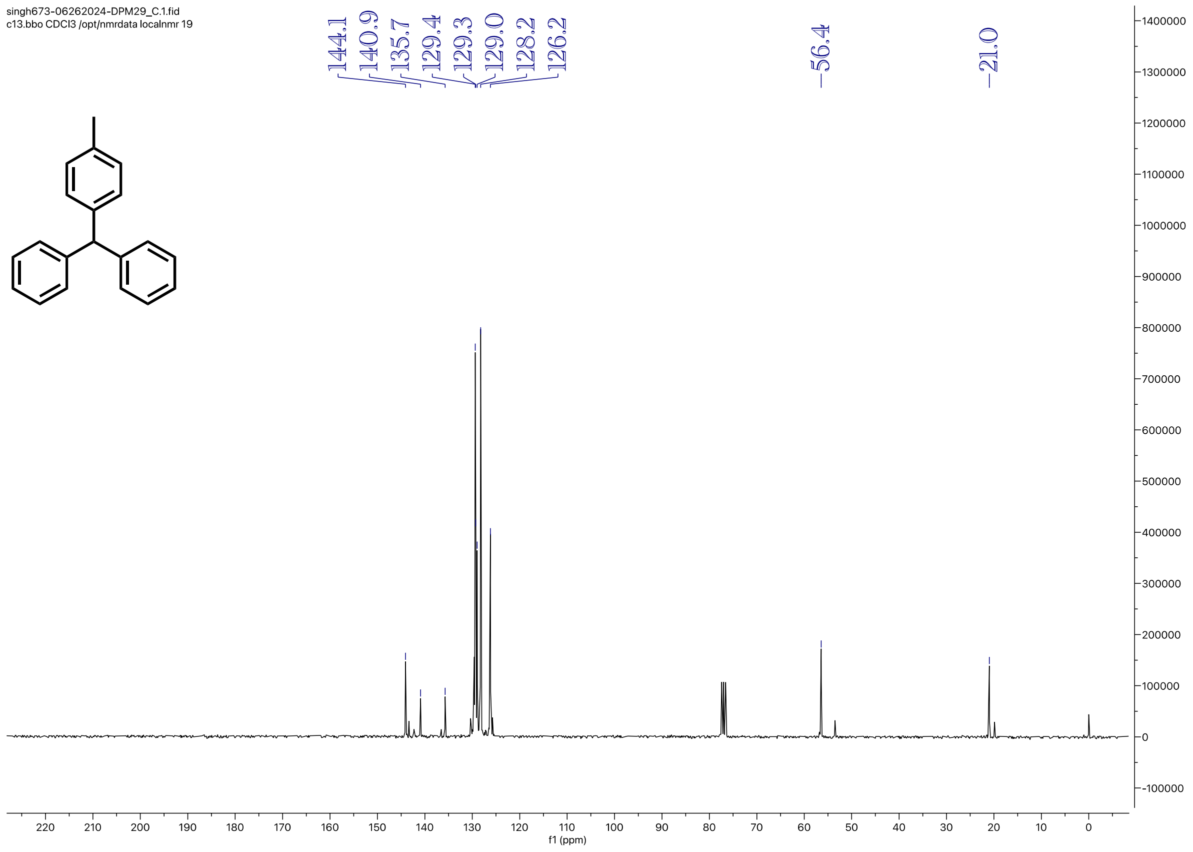

^{13}C {^{1}H} NMR (75 MHz, Chloroform-*d*) (*p*-tolylmethylene)dibenzene **(5)**
